# A Direct Method for β-Selective Glycosylation with an *N*-Acetylglucosamine Donor Armed by a 4-*O*-TBDMS Protecting Group

**DOI:** 10.3390/molecules22030429

**Published:** 2017-03-08

**Authors:** Hidenori Tanaka, Yu Hamaya, Hiyoshizo Kotsuki

**Affiliations:** 1 Oceanography Section, Science Research Center, Kochi University, Otsu, Nankoku-shi, Kochi 783-8502, Japan; 2 Laboratory of Natural Products Chemistry, Faculty of Science, Kochi University, Akebono-cho, Kochi-shi, Kochi 780-8520, Japan; a_paradise_country@yahoo.co.jp (Y.H.); kotsuki@kochi-u.ac.jp (H.K.)

**Keywords:** *N*-acetylglucosamine, β-selective glycosylation, remote protecting group effect, *O*-TBDMS protecting group

## Abstract

A new direct method for β-selective glycosylation with an *N*-acetylglucosamine (GlcNAc) donor was developed. This substrate, which can be readily prepared from commercially available GlcNAc in two steps, contains a 4-*O*-*tert*-butyldimethylsilyl (TBDMS) protecting group as a key component. We found that this functionality could have a favorable effect on the reactivity of the GlcNAc donor. Glycosylation with the armed donor using primary alcohols in the presence of a catalytic amount of trimethylsilyl trifluoromethanesulfonate (TMSOTf) in 1,2-dichloroethane smoothly gave the desired coupling products in good yields with complete β-selectivity, while sterically hindered acceptors were less efficient.

## 1. Introduction

**Figure 1:**
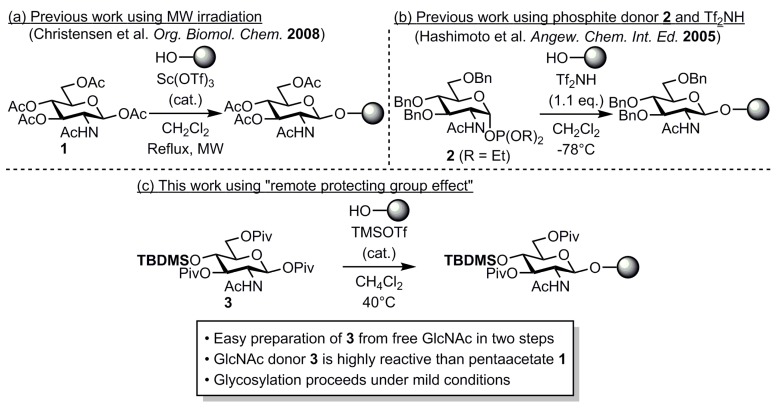
Glycosylation with GlcNAc donors: previous work (**a**,**b**); this work (**c**).

*N*-Acetylglucosamine (GlcNAc) is one of the most abundant naturally occurring monosaccharides, and it exists as a key component of oligosaccharides in glycoproteins and glycolipids that play important biological roles [1]. For example, erythropoietin [2] is an *N*-linked glycoprotein that enhances hematopoiesis. Several antibiotics, such as TMG-chitotriomycin [3] and bulgecins [4], are also glycosylated with GlcNAc. For the syntheses of glycoconjugates containing GlcNAc residues, it is necessary to use glycosamine (GlcN) donors activated by a phthaloyl [5] and 2,2,2-trichloroethoxycarbonyl [6,7] attached to the amine function. Unfortunately, however, methods using GlcNAc donors suffer from several disadvantages: (1) GlcNAc donors are less reactive than *N*-modified GlcN donors; (2) harsh conditions (reflux heating [8,9] and microwave irradiation [10,11]) are required to achieve the desired glycosylations; and (3) the corresponding oxazoline byproducts are formed in many cases. In 2008, Christensen and coworkers reported that scandium(III) trifluoromethanesulfonate (Sc(OTf)_3_) could serve as an effective activator for β-GlcNAc tetraacetate donor **1** and the glycosylation of simple alcohols in refluxing dichloromethane (Figure 1a) gave the desired β-glycosides in high yields [11]. With sterically hindered acceptors, disappointingly, the couplings resulted in low to moderate yields even when using excess amounts of **1** with microwave irradiation at 80 °C. On the other hand, Hashimoto et al. successfully developed glycosylations with a diethyl phosphite donor of GlcNAc **2** in the presence of stoichiometric bis(trifluoromethane)sulfonamide (Tf_2_NH) at −78 °C (Figure 1b), using sugar secondary alcohols, and then proceeded smoothly to obtain the disaccharides in good yields with complete β-selectivity [12]. However, this glycosylation method requires a stoichiometric amount of Tf_2_NH and cumbersome preparation of **2**. Accordingly, it would be desirable to develop a direct method for β-selective glycosylation with the use of readily available GlcNAc donors under mild conditions without the need for expensive rare-earth metal triflates and excess organic triflates.

Demchenko and coworkers demonstrated that thioglycoside donors of Glc were dramatically activated by the installation of electron-donating benzyl groups to both 4 and 6 positions, and disclosed the correlation between the stability of the glycosyl cations and the reactivity of the glycosyl donors, which led to the development of super-armed donors [13]. Takahashi and Toshima et al. reached a similar conclusion to activate 2,3-dideoxy Glc donors by introducing with a benzyl protecting group at either the 4 or 6 position [14]. This activation, which is called the “remote protecting group effect”, can be explained by considering that the presence of an electron-donating group at either the 4 or 6 position of Glc has a favorable stereoelectronic effect on the ring oxygen and facilitates the formation of an oxocarbenium ion intermediate by the elimination of a leaving group. We thought that the concept of this “remote protecting group effect” could also be valuable in dictating our glycosylation strategy. Based on these considerations, herein we examined a new direct method for β-selective glycosylation under mild conditions with GlcNAc donor **3** armed by a 4-*O*-*tert*-butyldimethylsilyl (TBDMS) protecting group (Figure 1c). Herein we describe the successful results of our implementation and the substrate scope of this synthetic sequence.

## 2. Results and Discussion

**Scheme 1:**
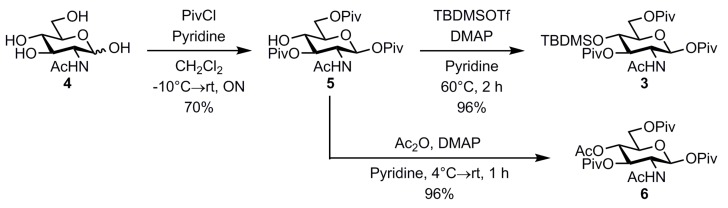
Preparation of GlcNAc donors **3** and **6**.

Following Ling’s previous report [15], β-GlcNAc tripivaloate **5** was easily prepared in 70% yield by the reaction of free GlcNAc **4** with pivaloyl chloride in pyridine and dichloromethane (Scheme 1). Next, we examined the installation of electron-donating groups at the 4 position of **5**. Unfortunately, however, all attempts to introduce a benzyl group using benzyl trichloroacetimidate [16] or 2,4,6-tris(benzyloxy)-1,3,5-triazine (TriBOT) [17] under acid catalysis failed. Not surprisingly, the steric hindrance around the 4-hydroxyl group was quite strong due to the presence of the neighboring bulky pivaloyl groups. To gain a similar “remote protecting group effect”, we examined the utility of TBDMS protection. Thus, the exposure of **5** to silylation with highly reactive *tert*-butyldimethylsilyl trifluoromethanesulfonate (TBDMSOTf) using 4-dimethylpyridine (DMAP) in pyridine [18] provided the corresponding 4-*O*-TBDMS-protected GlcNAc **3** in almost quantitative yield. The observed ring vicinal ^1^H-^1^H coupling constants (*J*_1,2_ = *J*_2,3_ = *J*_3,4_ = *J*_4,5_ = 8–9 Hz) show that the conformation of **3** would still be the ^4^C_1_ chair form. For comparison, we also prepared 4-*O*-acetylated derivative **6**.

**Scheme 2:**
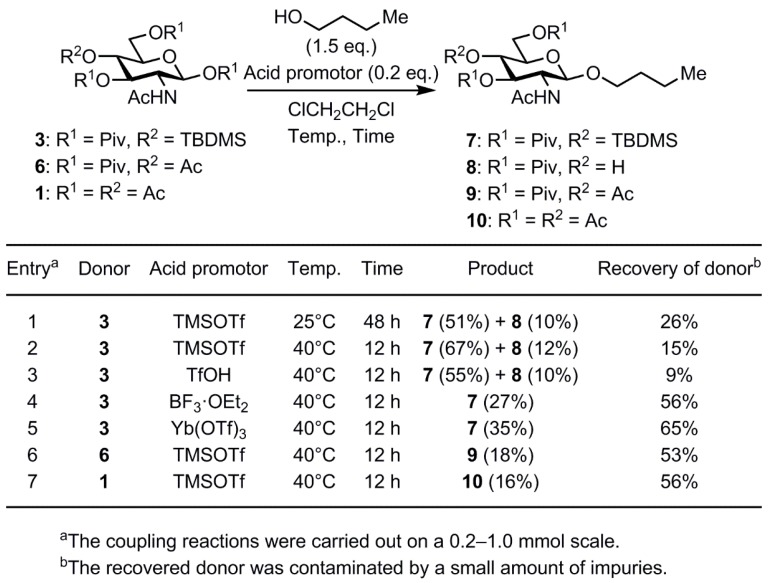
Optimization of reaction conditions during glycosylation with GlcNAc donors **1**, **3**, **6**.

To verify the “remote protecting group effect” by the 4-*O*-TBDMS group, we first examined glycosylation with GlcNAc donors **1**, **3**, and **6** using 1.5 equivalent of 1-butanol (Scheme 2). All reactions were carried out in 1,2-dichloroethane in the presence of a 20 mol % amount of a variety of acid promoters. When **3** was reacted with trimethylsilyl trifluoromethanesulfonate (TMSOTf) at 25 °C for 48 h, after work-up, the coupling product **7** was obtained in 51% yield with complete β-selectivity, accompanied by the formation of 10% of TBDMS-deprotected **8** along with 26% recovery of the starting material **3** (Entry 1). As expected, at 40 °C, the TMSOTf-catalyzed glycosylation was completed within 12 h to afford **7** and **8** in respective 67% and 12% yields (Entry 2). In contrast, the use of trifluoromethanesulfonic acid (TfOH) resulted in a slight decrease in the product yield, which indicated that TfOH was less efficient, probably due to its strong acidity and/or scarce solubility in this medium. We found that other Lewis acids such as boron trifluoride diethyl etherate (BF_3_•OEt_2_) and ytterbium(III) trifluoromethanesulfonate (Yb(OTf)_3_) were not as effective for our purpose (Entries 4 and 5). In all of these examples, the corresponding oxazoline by-product was detected in a trace amount. Under the conditions of Entry 2, as expected, glycosylation with 4-*O*-acetylated donor **6** dramatically reduced the coupling yield to 18% (Entry 6). Compared with **6**, β-GlcNAc tetraacetate donor **1** also gave only 16% of **10**, indicating the size of the acyloxy leaving group had no effect on the reaction efficiency (Entry 7). These experiments suggested that the presence of an electron-donating TBDMS group at the 4 position of GlcNAc was quite effective for promoting the desired β-glycosylation under mild conditions due to its increased reactivity.

**Scheme 3:**
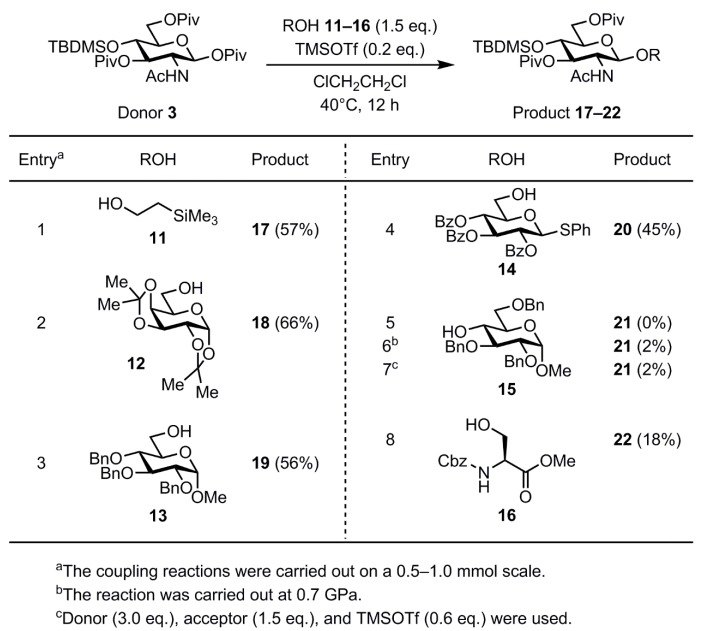
Substrate scope in glycosylation with GlcNAc donor **3**.

With the optimized conditions in hand, we next investigated the substrate scope in glycosylation with the armed donor **3** as shown in Scheme 3. Glycosylation of 2-(trimethylsilyl)ethanol **11** gave glycoside **17** in 58% yield (Entry 1). Using primary alcohols of **12** and **13**, the coupling products **18** and **19** were obtained in 66% and 56% yields, respectively (Entries 2 and 3). Interestingly, the reaction of **3** with thioglycoside Glc acceptor **14** proceeded successfully to give product **20**, a convenient building block in oligosaccharide synthesis, in 45% yield without aglycon transfer [19] (Entry 4). Despite several attempts to obtain GlcNAc-β(1→4)-Glc disaccharide **21**, a sterically hindered secondary alcohol of Glc **15** was not glycosylated even under high pressure conditions [20] (Entry 6) or with an excess amount of **3** (Entries 5–7). Finally, the coupling with a less reactive primary alcohol **16** of *N*-Cbz–protected l-serine provided the desired product **22** in only 18% yield (Entry 8).

## 3. Experimental Section

### 3.1. General Methods

^1^H- and ^13^C-NMR spectra were recorded with a JEOL ECA-500 (JEOL Ltd., Tokyo, Japan). High resolution mass spectrometry (HRMS) was performed with a Bruker Daltonics micrOTOF (ESI-TOF, Bruker Corp., Billerica, MA, USA). Specific optical rotation was recorded with a JASCO P-2200 (JASCO Corp., Tokyo, Japan). All reaction solvents were pre-dried with MS4 Å before use. Thin layer chromatography was performed using Merck TLC silica gel 60F_254_ on glass (Darmstadt, Germany). Developed TLC plates were stained with UV light (254 nm) and p-anisaldehyde solution. High pressure experiments were performed with a LECO PG-200-HPC (LECO Corp., St. Joseph, MI, USA). Purification was carried out by flash column chromatography (Silica Gel 60 N, 40–50 μm, Kanto Chemical Co., Inc., Tokyo, Japan) and gel filtration (Sephadex LH-20, GE Healthcare Bio-Science AB, Uppsala, Sweden). NMR spectra for all new compounds are available in Supplementary Materials.

### 3.2. General Experimental Procedure and Physical Data for All New Compounds

*2-Acetamido-2-deoxy-1,3,6-tri-O-pivaloyl-4-O-tert-butyldimethylsilyl-β-d-glucopyranoside* (**3**). To a solution of 2-acetamido-2-deoxy-1,3,6-di-*O*-pivaloyl-β-d-glucopyranoside **3** (4.73 g, 10.0 mmol) and 4-dimethylaminopyridine (244 mg, 2.0 mmol) in pyridine (10.0 mL) was added *tert*-butyldimethylsilyl trifluoromethanesulfonate (2.7 mL, 12.0 mmol) at 4 °C under a nitrogen atmosphere. The reaction was warmed up to 60 °C and stirred at the same temperature for 2 h. After the reaction was complete, the mixture was cool to room temperature and evaporated with toluene (20 mL × 3). The residue was dissolved in ethyl acetate (200 mL) and washed successively with 2.0 M HCl aq. (200 mL), satd. NaHCO_3_ aq. (200 mL × 2), and brine (200 mL). The aqueous layers were back-extracted with ethyl acetate (200 mL × 2). The combined extracts were dried over Na_2_SO_4_, filtered off, and concentrated in vacuo. The crude product was purified by flash column chromatography (ethyl acetate:hexane = 1:4–1:2) to give 2-acetamido-2-deoxy-1,3,6-tri-*O*-pivaloyl-4-*O*-*tert*-butyldimethylsilyl-β-d-glucopyranoside as a colorless syrup (5.62 g, 96%). [α]^D^ +3.17° (*c* 1.69, CHCl_3_); ^1^H-NMR (500 MHz, CDCl_3_) δ 5.90 (d, 1H, *J* = 10.3 Hz, NH), 5.55 (d, 1H, *J* = 8.0 Hz, H-1), 5.08 (t, 1H, *J* = 8.9 Hz, H-3), 4.47 (dd, 1H, *J* = 2.6 Hz, *J* = 11.7 Hz, H-6a), 4.30 (q, 1H, *J* = 9.6 Hz, H-2), 4.11 (dd, 1H, *J* = 4.0 Hz, *J* = 12.0 Hz, H-6b), 3.92 (t, 1H, *J* = 8.3 Hz, H-4), 3.65 (m, 1H, H-5), 1.86 (s, 3H, Ac), 1.22, 1.21 and 1.17 (s, 27H, 3Piv), 0.85 (s, 9H, ^t^Bu), 0.097 and 0.085 (2s, 6H, SiMe_2_); ^13^C-NMR (125.8 MHz, CDCl_3_) δ 179.5, 178.0, 177.0, 169.4, 92.6, 75.2, 68.5, 62.2, 52.5, 39.3, 38.9, 38.7, 27.4, 27.2, 26.7, 25.7, 23.3, 18.0, −4.2, −4.6; HRMS (ESI-TOF) *m*/*z*: found [M + Na]^+^ 610.3381, C_29_H_53_NO_9_Si calcd. for [M + Na]^+^ 610.3382.

*2-Acetamido-4-O-acetyl-2-deoxy-1,3,6-tri-O-pivaloyl-β-d-glucopyranoside* (**6**). To a solution of 2-acetamido-2-deoxy-1,3,6-tri-*O*-pivaloyl-β-d-glucopyranoside **3** (4.75 g, 10.0 mmol) and 4-dimethylaminopyridine (241 mg, 2.0 mmol) in pyridine (10.0 mL) was added acetic anhydride (1.1 mL, 12.0 mmol) at 4 °C under a nitrogen atmosphere. The reaction was warmed up to room temperature and stirred for 1 h. After evaporation with toluene (20 mL × 3), the residue was dissolved in ethyl acetate (200 mL) and washed successively with 2.0 M HCl aq. (200 mL), satd. NaHCO_3_ aq. (200 mL × 2), and brine (200 mL). The aqueous layers were back-extracted with ethyl acetate (200 mL × 2). The combined extracts were dried over Na_2_SO_4_, filtered off, and concentrated in vacuo. The crude product was purified by flash column chromatography (ethyl acetate:hexane = 1:3 to 2:3) to give 2-acetamido-4-*O*-acetyl-2-deoxy-1,3,6-tri-*O*-pivaloyl-β-d-glucopyranoside as a colorless syrup (4.95 g, 96%). [α]^D^ +12.47° (*c* 3.84, CHCl_3_); ^1^H-NMR (500 MHz, CDCl_3_) δ 6.21 (d, 1H, *J* = 10.3 Hz, NH), 5.60 (d, 1H, *J* = 8.6 Hz, H-1), 5.21(m, 2H, *J* = 9.0 Hz, H-3 and H-4), 4.44 (q, 1H, *J* = 9.8 Hz, H-2), 4.19 (m, 2H, H-6a and H-6b), 3.87 (m, 1H, H-5), 2.04 (s, 3H, Ac), 1.88(s, 3H, Ac), 1.22, 1.19 and 1.15 (3s, 27H, 3Piv); ^13^C-NMR (125.8 MHz, CDCl_3_) δ 179.3, 178.1, 176.9, 169.5, 168.9, 92.5, 72.7, 72.5, 67.9, 61.6, 52.3, 38.9, 38.8, 38.7, 27.0, 26.8, 26.7, 22.9, 20.5; HRMS (ESI-TOF) *m*/*z*: found [M + Na]^+^ 538.2616, C_25_H_41_NO_10_Si calcd. for [M + Na]^+^ 538.2623.

### 3.3. General Procedure for the Glycosylation with GlcNAc Donors

To a mixture of GlcNAc donor (1.00 mmol) and acceptor (1.50 mmol) in 1,2-dichloroethane (5.0 mL) was added TMSOTf (36 μL, 0.2 mmol) at 40 °C. After the reaction was stirred at the same temperature for 12 h, it was quenched by the addition of satd. aq. NaHCO_3_ (50 mL). The resulting mixture was then extracted with CHCl_3_ (50 mL × 3) and washed with brine (50 mL). The combined extracts were dried over Na_2_SO_4_, filtered off, and concentrated in vacuo. The crude product was purified by flash column chromatography to give the desired GlcNAc glycoside. Additional purification using Sephadex LH-20 (MeOH/CHCl_3_ = 1:1) was performed if needed.

*Butyl 2-acetamido-2-deoxy-3,6-di-O-pivaloyl-4-O-tert-butyldimethylsilyl-β-d-glucopyranoside* (**7**). [α]^D^ −12.28° (*c* 1.49, CHCl_3_); ^1^H-NMR (500 MHz, CDCl_3_) δ 6.09 (d, 1H, *J* = 9.8 Hz, NH), 5.02 (t, 1H, *J* = 8.3 Hz, H-3^GlcNAc^), 4.54 (dd, 1H, *J* = 3.7 Hz, *J* = 11.7 Hz, H-6a^GlcNAc^), 4.41 (d, 1H, *J* = 6.9 Hz, H-1^GlcNAc^), 4.10 (m, 2H, H-6b^GlcNAc^ and H-2^GlcNAc^), 3.85 (t, 1H, *J* = 7.5 Hz, H-4^GlcNAc^), 3.80 (m, 1H, OC*Ha*Hb(CH_2_)_2_CH_3_), 3.59 (m, 1H, H-5^GlcNAc^), 3.40 (m, 1H, OCHa*Hb*(CH_2_)_2_CH_3_), 1.92 (s, 3H, Ac), 1.44 (m, 2H, OCH_2_C*H*_2_CH_2_CH_3_), 1.25 (m, 2H, O (CH_2_)_2_ C*H*_2_CH_3_), 1.23 and 1.21 (2s, 18H, 2Piv), 0.88 (t, 3H, *J* = 6.8 Hz, O(CH_2_)_3_C*H*_3_), 0.85 (s, 9H, ^t^Bu), 0.12 and 0.093 (2s, 6H, SiMe_2_); ^13^C-NMR (125.8 MHz, CDCl_3_) δ 179.1, 178.0, 169.5, 100.7, 74.4, 74.3, 68.8, 68.4, 62.9, 52.5, 39.2, 38.8, 31.4, 27.3, 27.2, 25.7, 23.4, 19.0, 18.0, 13.7, −4.3, −4.7; HRMS (ESI-TOF) *m*/*z*: found [M + Na]^+^ 582.3432, C_28_H_53_NO_8_Si calcd. for [M + Na]^+^ 582.3433.

*Butyl 2-acetamido-2-deoxy-4-hydroxy-3,6-di-O-pivaloyl-β-d-glucopyranoside* (**8**). [α]^D^ −40.52° (*c* 3.83, CHCl_3_); ^1^H-NMR (500 MHz, CDCl_3_) δ 6.40 (m, 1H, NH), 5.13 (t, 1H, *J* = 9.7 Hz, H-3^GlcNAc^), 4.47 (d, 1H, *J* = 8.6 Hz, H-1^GlcNAc^), 4.36 (dd, 1H, *J* = 2.1 Hz, *J* = 11.7 Hz, H-6a^GlcNAc^), 4.24 (dd, 1H, *J* = 5.7 Hz, *J* = 12.1 Hz, H-6b^GlcNAc^), 3.86 (q, 1H, *J* = 9.3 Hz, H-2^GlcNAc^), 3.75 (m, 1H, OC*Ha*Hb(CH_2_)_2_CH_3_), 3.55 (m, 1H, H-5^GlcNAc^), 3.47 (m, 1H, OH), 3.44 (m, 1H, H-4^GlcNAc^), 3.39 (m, 1H, OCHa*Hb*(CH_2_)_2_CH_3_), 1.84 (s, 3H, Ac), 1.53–1.40 (m, 2H, OCH_2_C*H*_2_CH_2_CH_3_), 1.31–1.21 (m, 2H, O (CH_2_)_2_C*H*_2_CH_3_), 1.15 and 1.13 (2s, 18H, 2Piv), 0.82 (t, 3H, *J* = 7.5 Hz, O(CH_2_)_3_C*H*_3_); ^13^C-NMR (125.8 MHz, CDCl_3_) δ 179.8, 179.0, 170.1, 100.8, 75.1, 74.0, 69.6, 69.1, 63.5, 53.9, 38.9, 38.8, 31.3, 27.1, 27.0, 23.0, 18.9, 13.6; HRMS (ESI-TOF) *m*/*z*: found [M + Na]^+^ 468.2567, C_28_H_53_NO_8_Si calcd. for [M + Na]^+^ 468.2568.

*Butyl 2-acetamido-4-O-acetyl-2-deoxy-3,6-di-O-pivaloyl-β-d-glucopyranoside* (**9**). [α]^D^ −8.91° (*c* 2.875, CHCl_3_); ^1^H-NMR (500 MHz, CDCl_3_) δ 6.03 (m, 1H, NH), 5.22 (t, 1H, *J* = 10.0 Hz, H-3^GlcNAc^), 5.11 (t, 1H, *J* = 9.7 Hz, H-4^GlcNAc^), 4.57 (d, 1H, *J* = 8.1 Hz, H-1^GlcNAc^), 4.20 (dd, 1H, *J* = 2.3 Hz, *J* = 12.0 Hz, H-6a^GlcNAc^), 4.15 (dd, 1H, *J* = 5.7 Hz, *J* = 12.1 Hz, H-6b^GlcNAc^), 4.06 (q, 1H, *J* = 9.5 Hz, H-2^GlcNAc^), 3.84 (m, 1H, OC*Ha*Hb(CH_2_)_2_CH_3_), 3.74 (m, 1H, H-5^GlcNAc^), 3.48 (m, 1H, OCHa*Hb*(CH_2_)_2_CH_3_), 2.01 and 1.92 (2s, 6H, 2Ac), 1.61–1.49 (m, 2H, OCH_2_C*H*_2_CH_2_CH_3_), 1.34 (m, 2H, O (CH_2_)_2_C*H*_2_CH_3_), 1.22 and 1.15 (2s, 18H, 2Piv), 0.89 (t, 3H, *J* = 7.3 Hz, O(CH_2_)_3_C*H*_3_); ^13^C-NMR (125.8 MHz, CDCl_3_) δ 178.6, 178.1, 169.8, 169.0, 100.9, 72.2, 71.8, 69.1, 68.5, 62.3, 53.9, 38.8, 38.7, 31.3, 27.0, 26.8, 23.0, 20.5, 18.9, 13.6; HRMS (ESI-TOF) *m*/*z*: found [M + Na]^+^ 510.2676, C_28_H_53_NO_8_Si calcd. for [M + Na]^+^ 510.2674.

*2-Trimethylsilylethyl 2-acetamido-2-deoxy-3,6-di-O-pivaloy-4-O-tert-butyldimethylsilyl-β-d-glucopyranoside* (**17**). [α]^D^ −16.51°(*c* 3.465, CHCl_3_); ^1^H-NMR (500 MHz, CDCl_3_) δ 6.26 (d, 1H, *J* = 9.8 Hz, NH^GlcNAc^), 5.04 (t, 1H, *J* = 8.6 Hz, H-3^GlcNAc^), 4.49 (dd, 1H, *J* = 1.95 Hz, *J* = 11.5 Hz, H-6a^GlcNAc^), 4.36 (d, 1H, *J* = 6.9 Hz, H-1^GlcNAc^), 4.05 (m, 2H, H-2^GlcNAc^, H-6b^GlcNAc^), 3.85 (m, 1H, OC*Ha*HbCH_2_SiMe_3_), 3.81 (t, 1H, *J* = 8.0 Hz, H-4^GlcNAc^), 3.51 (m, 1H, H-5^GlcNAc^), 3.45 (m, 1H, OCHa*Hb*CH_2_SiMe_3_), 1.88 (s, 3H, Ac), 1.19 and 1.17 (2s, 18H, 2Piv), 0.83 (m, 2H, OCH_2_C*H*_2_SiMe_3_), 0.82 (s, 9H, ^t^Bu), 0.075 and 0.044 (2s, 6H, SiMe_2_), −0.055 (s, 9H, SiMe_3_); ^13^C-NMR (125.8 MHz, CDCl_3_) δ 179.4, 178.0, 169.5, 100.2, 74.2, 68.8, 66.4, 62.8, 53.0, 39.2, 38.8, 27.3, 27.2, 25.7, 23.5, 18.0, 17.8, −1.5, −4.2, −4.6; HRMS (ESI-TOF) *m*/*z*: found [M + Na]^+^ 626.3525, C_29_H_57_NO_8_Si_2_ calcd. for [M + Na]^+^ 626.3515.

*2-Acetamido-2-deoxy-3,6-di-O-pivaloyl-4-O-tert-butyldimethylsilyl-β-d-glucopyranosyl-(1→6)-1,2:3,4-di-O-isopropylidene-α-d-galactopyranose* (**18**). [α]^D^ +0.22° (*c* 1.04, CHCl_3_); ^1^H-NMR (500 MHz, CDCl_3_) δ 5.63 (d, 1H, *J* = 9.8 Hz, NH^GlcNAc^), 5.50 (d, 1H, *J* = 5.2 Hz, H-1^Gal^), 4.99 (t, 1H, *J* = 8.9 Hz, H-3^GlcNAc^), 4.56 (dd, 1H, *J* = 2 Hz, *J* = 8 Hz, H-3^Gal^), 4.54 (d, 1H, *J* = 3 Hz, H-1^GlcNAc^), 4.52 (dd, 1H, *J* = 3.3 Hz, *J* = 4.3 Hz, H-6a^GlcNAc^), 4.29 (m, 1H, H-2^Gal^), 4.11 (m, 3H, H-6b^GlcNAc^, H-4^Gal^ and H-2^GlcNAc^), 3.92 (m, 2H, H-5^Gal^ and H-6a^Gal^), 3.84 (t, 1H, *J* = 8 Hz, H-4^GlcNAc^), 3.67 (dd, 1H, *J* = 8.85 Hz, *J* = 12.9 Hz, H-6b^Gal^), 3.55 (m, 1H, H-5^GlcNAc^), 1.94 (s, 3H, Ac), 1.49 (s, 3H, COOMe_2_a), 1.42 (s, 3H, COOMe_2_a), 1.31 (s, 3H, COOMe_2_b), 1.30 (s, 3H, COOMe_2_b), 1.23 and 1.20 (2s, 18H, 2Piv), 0.844 (s, 9H, ^t^Bu), 0.093 and 0.064 (2s, 6H, SiMe_2_); ^13^C-NMR (125.8 MHz, CDCl_3_) δ 179.1, 178.0, 170.0, 109.2, 108.5, 101.8, 96.2, 75.1, 74.5, 71.1, 70.6, 70.2, 68.9, 68.2, 68.1, 62.7, 53.2, 39.2, 38.8, 27.4, 27.2, 26.1, 25.9, 25.7, 24.9, 23.5, 18.0, −4.2, −4.6; HRMS (ESI-TOF) *m*/*z*: found [M + Na]^+^ 768.3954, C_36_H_63_NO_13_Si calcd. for [M + Na]^+^ 768.3961.

*Methyl (2-acetamido-2-deoxy-3,6-di-O-pivaloyl-4-O-tert-butyldimethylsilyl-β-d-glucopyranosyl)-(1→6)-2,3,4-tri-O-benzyl-α-d-glucopyranoside* (**19**). [α]^D^ +1.70° (*c* 1.49, CHCl_3_); ^1^H-NMR (500 MHz, CDCl_3_) δ 7.30 (m, 15H, 3Ph), 5.70 (d, 1H, *J* = 9.2 Hz, NH^GlcNAc^), 4.98 and 4.80 (2d, 2H, *J* = 11 Hz, O*CH*_2*a*_Ph), 4.96 (t, 1H, *J* = 7.8 Hz, H-3^GlcNAc^), 4.84 and 4.56 (2d, 2H, *J* = 11 Hz, O*CH*_2*b*_Ph), 4.77 and 4.66 (2d, 2H, *J* = 12 Hz, O*CH*_2*c*_Ph), 4.53 (d, 1H, *J* = 7.0 Hz, H-1^Glc^), 4.49 (dd, 1H, *J* = 3.8 Hz, *J* = 11.9 Hz, H-6a^GlcNAc^), 4.39 (d, 1H, *J* = 6.9 Hz, H-1^GlcNAc^), 4.11 (dd, 1H, *J* = 7.5 Hz, *J* = 11.8 Hz, H-6b^GlcNAc^), 4.08 (q, 1H, *J* = 9.0 Hz, H-2^GlcNAc^), 4.02 (dd, 1H, *J* = 1.5 Hz, *J* = 10.5 Hz, H-2^Glc^), 3.97 (t, 1H, *J* = 9.5 Hz, H-3^Glc^), 3.82 (t, 1H, *J* = 7.4 Hz, H-4^GlcNAc^), 3.72 (dd, 1H, *J* = 2.3 Hz, *J* = 9.3 Hz, H-5^Glc^), 3.60 (m, 2H, H-6a^Glc^ and H-5^GlcNAc^), 3.48 (dd, 1H, *J* = 3.8 Hz, *J* = 10.3 Hz, H-6b^Glc^), 3.46 (t, 1H, *J* = 10 Hz, H-4^Glc^), 3.35 (s, 3H, OMe), 1.83 (s, 3H, Ac), 1.19 and 1.16 (2s, 18H, 2Piv), 0.86 (s, 9H, ^t^Bu), 0.097 and 0.074 (2s, 6H, SiMe_2_); ^13^C-NMR (125.8 MHz, CDCl_3_) δ178.8, 177.9, 169.3, 138.8 ,138.2, 138.1, 128.43, 128.39, 128.3, 128.1, 127.8, 127.77, 127.5, 100.9, 97.8, 82.0, 79.6, 77.4, 75.6, 74.7, 74.5, 73.9, 73.2, 69.5, 68.3, 67.3, 63.0, 55.0, 52.5, 39.1, 38.8, 27.2, 27.1, 25.7, 23.4, 17.9, −4.3, −4.7; HRMS (ESI-TOF) *m*/*z*: found [M + Na]^+^ 972.4902, C_52_H_75_NO_13_Si calcd. for [M + Na]^+^ 972.4900.

*Phenyl (2-acetamido-2-deoxy-3,6-di-O-pivaloyl-4-O-tert-butyldimethylsilyl-β-d-glucopyranosyl)-(1→6)-2,3,4-tri-O-benzoyl-1-thio-β-d-glucopyranoside* (**20**). [α]^D^ −0.14° (*c* 2.06, CHCl_3_); ^1^H-NMR (500 MHz, CDCl_3_) δ 7.96–7.23 (20H, 4Ph), 5.86 (t, 1H, *J* = 9.5 Hz, H-3^Glc^), 5.62 (d, 1H, *J* = 9.8 Hz, NH^GlcNAc^), 5.44 (t, 1H, *J* = 9.7 Hz, H-4^Glc^), 5.39 (t, 1H, *J* = 9.7 Hz, H-2^Glc^), 5.00 (m, 2H, H-3^GlcNAc^ and H-1^Glc^), 4.51 (dd, 1H, *J* = 2.6 Hz, *J* = 11.7 Hz, H-6a^GlcNAc^), 4.36 (d, 1H, *J* = 8.0 Hz, H-1^GlcNAc^), 4.11–4.02 (m, 3H, H-2^GlcNAc^, H-6a^Glc^ and H-6b^GlcNAc^), 3.97 (m, 1H, H-5^Glc^), 3.82 (t, 1H, *J* = 8.6 Hz, H-4^GlcNAc^), 3.62 (dd, 1H, *J* = 6.3 Hz, *J* = 12.0 Hz, H-6b^Glc^), 3.46 (m, 1H, H-5^GlcNAc^), 1.86 (s, 3H, Ac), 1.23 and 1.20 (2s, 18H, 2Piv), 0.85 (s, 9H, ^t^Bu), 0.093 and 0.070 (2s, 6H, SiMe_2_); ^13^C-NMR (125.8 MHz, CDCl_3_) δ 178.7, 178.0, 170.0, 165.6, 165.4, 164.9, 133.6, 133.4, 133.3, 133.2, 131.5, 129.9, 129.8, 129.6, 129.3, 129.1, 128.7, 128.5, 128.3, 128.2, 101.8, 86.2, 78.1, 74.9, 74.6, 74.2, 70.3, 68.9, 68.9, 67.6, 62.5, 53.5, 39.2, 38.8, 27.4, 27.2, 25.7, 23.3, 18.0, −4.1, −4.6; HRMS (ESI-TOF) *m*/*z*: found [M + Na]^+^ 1092.4225, C_52_H_75_NO_13_Si calcd. for [M + Na]^+^ 1092.4206.

*Methyl (2-acetamido-2-deoxy-3,6-di-O-pivaloyl-4-O-tert-butyldimethylsilyl-β-d-glucopyranosyl)-(1→4)-2,3,6-tri-O-benzyl-α-d-glucopyranoside* (**21**). [α]^D^ −4.12°(*c* 0.98, CHCl_3_); ^1^H-NMR (500 MHz, CDCl_3_) δ 7.53–7.24 (m, 15H, 3Ph), 4.96 and 4.72 (2d, 2H, *J* = 11.5 Hz, O*CH*_2*a*_Ph), 4.86 and 4.30 (2d, 2H, *J* = 12.3 Hz, O*CH*_2*b*_Ph), 4.67 and 4.54 (2d, 2H, *J* = 12.3 Hz, O*CH_2_c*Ph), 4.66 (t, 1H, *J* = 9.5 Hz, H-3^GlcNAc^), 4.54 (d, 1H, *J* = 3.4 Hz, H-1^Glc^), 4.46 (d, 1H, *J* = 10.3 Hz, NH^GlcNAc^), 4.40 (dd, 1H, *J* = 2.0 Hz, *J* = 11.7 Hz, H-6a^GlcNAc^), 4.05 (d, 1H, *J* = 8.6 Hz, H-1^GlcNAc^), 3.87 (q, 1H, *J* = 9.4 Hz, H-2^GlcNAc^), 3.81 (t, 1H, *J* = 9.2 Hz, H-6a^GlcNAc^), 3.75 (t, 1H, *J* = 9.5 Hz, H-6b^GlcNAc^), 3.65 (dd, 1H, *J* = 6.9 Hz, *J* = 11.5 Hz, H-4^Glc^), 3.60 (m, 1H, H-5^GlcNAc^), 3.59 (dd, 1H, *J* = 3.6 Hz, *J* = 10.5 Hz, H-6a^Glc^), 3.55 (t, 1H, *J* = 8.9 Hz, H-4^GlcNAc^), 3.44 (dd, 1H, *J =* 1.3 Hz, *J* = 10.4 Hz, H-6b^Glc^), 3.39–3.33 (m, 5H, H-3^Glc^, H-2^Glc^, OMe), 3.29 (m, 1H, H-5^Glc^), 1.68 (s, 3H, Ac), 1.17 (2s, 18H, 2Piv), 0.87 (s, 9H, ^t^Bu), 0.053 and 0.019 (2s, 6H, SiMe_2_); ^13^C-NMR (125.8 MHz, CDCl_3_) δ 178.7, 178.0, 169.2, 139.7, 138.3, 137.5, 129.4, 129.2, 129.1, 129.0, 128.3, 128.1, 128.0, 127.9, 127.8, 127.7, 127.5, 127.0, 100.7, 98.6, 79.6, 78.2, 75.9, 75.0, 73.9, 73.8, 73.4, 69.6, 69.4, 67.1, 63.3, 55.3, 54.3, 39.1, 38.8, 27.4, 27.2, 27.0, 25.7, 23.4, 18.0, −4.1, −4.4; HRMS (ESI-TOF) *m*/*z*: found [M + Na]^+^ 972.4917, C_52_H_75_NO_13_Si calcd. for [M + Na]^+^ 972.4900.

*N-(Benzyloxycarbonyl) 3-O-(2-acetamido-2-deoxy-3,6-di-O-pivaloyl-4-O-tert-butyldimethylsilyl-β-d-glucopyranosyl)-l-serine methyl ester* (**22**). [α]^D^ −4.89° (*c* 2.13, CHCl_3_); ^1^H-NMR (500 MHz, CDCl_3_) δ 7.34 (m, 5H, Ph), 5.94 (m, 1H, NH^GlcNAc^), 5.78 (d, 1H, *J* = 8.6 Hz, NH^Ser^), 5.13 and 5.09 (2d, 2H, *J* = 12.3 Hz, *CH*_2_Ph), 5.00 (t, 1H, *J* = 8.9 Hz, H-3^GlcNAc^), 4.48 (m, 2H, H-6a^GlcNAc^ and H-2^Ser^), 4.39 (d, 1H, *J* = 7.4 Hz, H-1^GlcNAc^), 4.16 (dd, 1H, *J =* 3.8 Hz, *J* = 10.8 Hz, H-3a^Ser^), 4.07 (dd, 1H, *J* = 4.5 Hz, *J* = 11.5 Hz, H-6b^GlcNAc^), 4.02 (q, 1H, *J* = 8.8 Hz, H-2^GlcNAc^), 3.83 (t, 1H, *J* = 8.3 Hz, H-4^GlcNAc^), 3.78 (dd, 1H, *J* = 3.5 Hz, *J* = 10.5 Hz, H-3b^Ser^), 3.73 (s, 3H, OMe), 3.50 (m, 1H, H-5^GlcNAc^), 1.84 (s, 3H, Ac), 1.22 and 1.18 (s, 18H, 2Piv), 0.84 (s, 9H, ^t^Bu), 0.092 and 0.061 (2s, 6H, SiMe_2_); ^13^C-NMR (125.8 MHz, CDCl_3_) δ 179.2, 177.9, 170.2, 170.1, 156.0, 136.2, 128.4, 128.1, 101.0, 74.5, 74,49, 68.4, 68.0, 67.0, 62.3, 53.9, 53.0, 52.6, 39.2, 38.8, 27.3, 27.2, 23,3, 18.0, −4.3, −4.7; HRMS (ESI-TOF) *m*/*z*: found [M + Na]^+^ 761.3651, C_36_H_58_N_2_O_12_Si calcd. for [M + Na]^+^ 761.3651.

## 4. Conclusions

In summary, based on the concept of the “remote protecting group effect”, we developed a direct method for β-selective glycosylation under mild conditions with GlcNAc donor **3** bearing a TBDMS protecting group at the 4 position. Thus, the attachment of this functionality could improve the reactivity of **3** compared with that of β-GlcNAc tetraacetate **1**. Furthermore, the substrate scope in this glycosylation revealed that, while primary alcohols gave the corresponding coupling products in good yields, sterically hindered alcohols were less efficient. Further studies to improve this glycosylation using GlcNAc donors are currently in progress in our laboratories.

## Supplementary Materials

The following are available online: NMR spectra for all new compounds.
