# Supplementary material for: A Direct Method for β-Selective Glycosylation with an N-Acetylglucosamine Donor Armed by a 4-O-TBDMS Protecting Group
Source: Molecules. 2017 Mar 8;22(3):429. doi: 10.3390/molecules22030429 (PMC6155425; doi:10.3390/molecules22030429)
Supplement: Supplementary file 1 [file molecules-22-00429-s001.pdf]

Supplementary data

for

A direct method for β-glycosylation with an *N*-acetylglucosamine aimed by a 4-*O*-TBDMS protecting group

Contents

NMR spectra of compound **3** -- p. 3–4

NMR spectra of compound **6** -- p. 5–6

NMR spectra of compound **7** -- p. 7–8

NMR spectra of compound **8** -- p. 9–10

NMR spectra of compound **9** -- p. 11–12

NMR spectra of compound **17** -- p. 13–14

NMR spectra of compound **18** -- p. 15–16

NMR spectra of compound **19** -- p. 17–18

NMR spectra of compound **20** -- p. 19–20

NMR spectra of compound **21** -- p. 21–22

NMR spectra of compound **22** -- p. 23–24

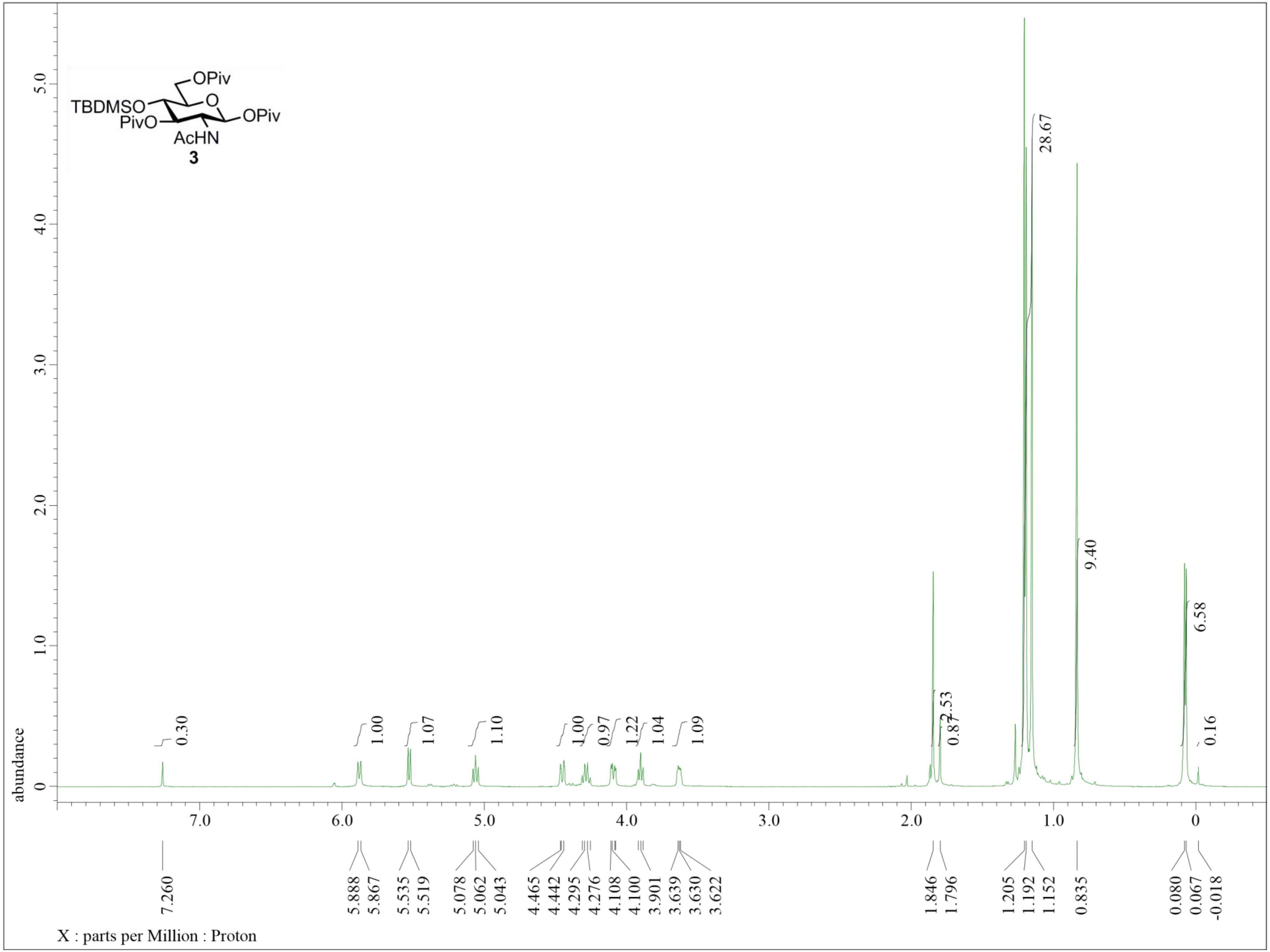
OPiv
TBDMSO
O
OPiv
PivO
AcHN
3
abundance
X : parts per Million : Proton
0.30
1.00
1.07
1.10
1.00
0.97
1.22
1.04
1.09
2.53
0.87
28.67
9.40
6.58
0.16
7.260
5.888
5.867
5.535
5.519
5.078
5.062
5.043
4.465
4.442
4.295
4.276
4.108
4.100
3.901
3.639
3.630
3.622
1.846
1.796
1.205
1.192
1.152
0.835
0.080
0.067
-0.018

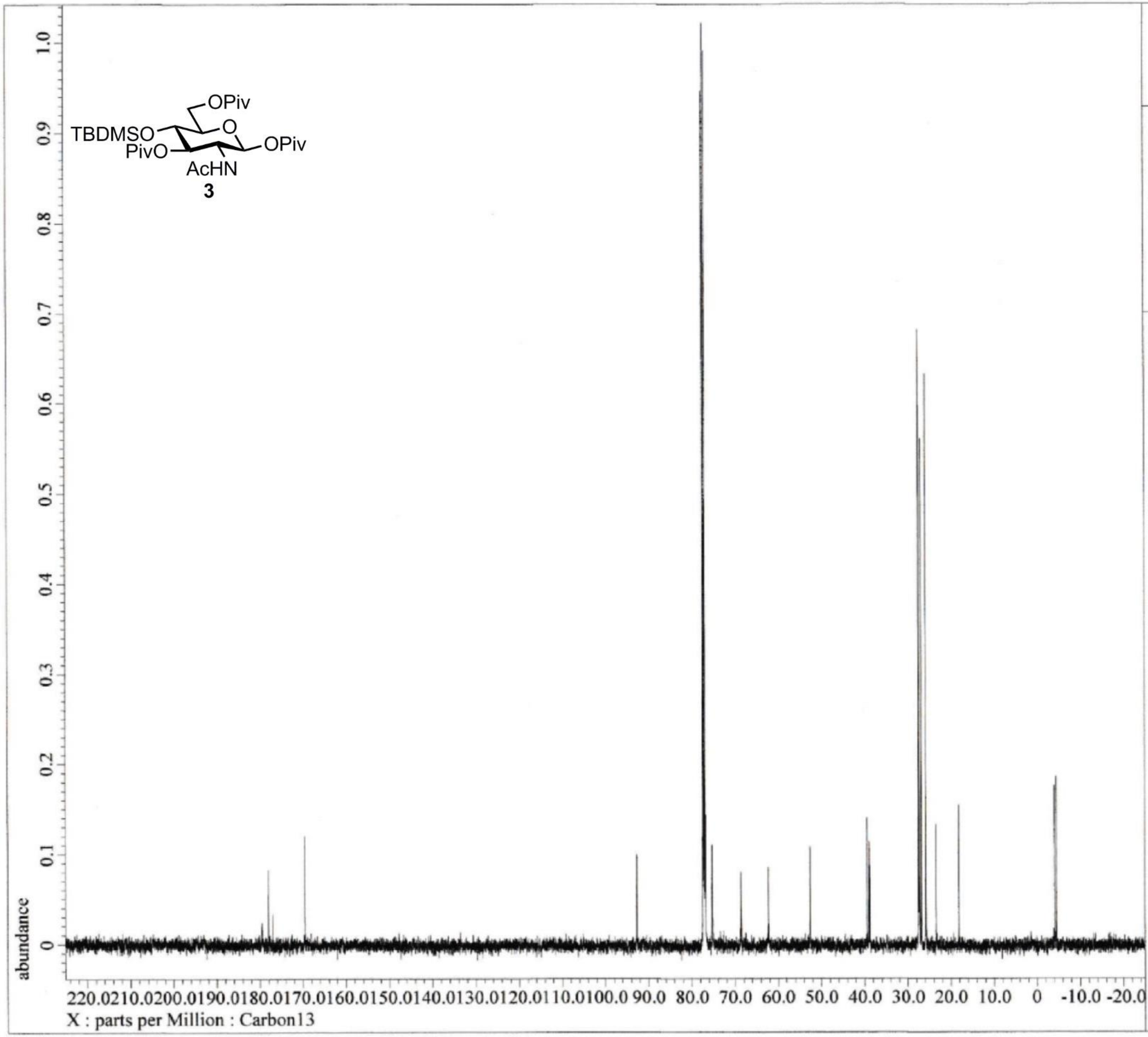

JEOL RESONANCE

```
---- PROCESSING PARAMETERS ----
dc_balance( 0, FALSE )
sexp( 2.0[Hz], 0.0[s] )
trapezoid( 0[%], 0[%], 80[%], 100[%] )
zerofill( 1 )
fft( 1, TRUE, TRUE )
machinephase
ppm

以下に由来:: NAc_4-Si_carbon-2-1.jdf

Filename          = NAc_4-Si_carbon-2-2.jdf
Author            = delta
Experiment        = carbon.jxp
Sample_Id         = NAc_4-Si
Solvent           = CHLOROFORM-D
Creation_Time     = 27-JUL-2015 10:51:47
Revision_Time     = 27-JUL-2015 11:03:06
Current_Time      = 27-JUL-2015 11:03:57

Comment           = single pulse decoupled gat
Data_Format       = 1D COMPLEX
Dim_Size          = 26214
Dim_Title         = Carbon13
Dim_Units         = [ppm]
Dimensions        = X
Site              = JMN-ECA500
Spectrometer      = DELTA2_NMR

Field_Strength    = 11.7473579[T] (500[MHz])
X_Acq_Duration    = 0.83361792[s]
X_Domain          = 13C
X_Freq            = 125.76529768[MHz]
X_Offset          = 100[ppm]
X_Points          = 32768
X_Prescans        = 4
X_Resolution      = 1.19959034[Hz]
X_Sweep           = 39.3081761[kHz]
X_Sweep_Clipped   = 31.44654088[kHz]
Irr_Domain        = Proton
Irr_Freq          = 500.15991521[MHz]
Irr_Offset        = 5.0[ppm]
Clipped           = FALSE
Scans             = 200
Total_Scans       = 200

Relaxation_Delay  = 2[s]
Recvr_Gain        = 56
Temp_Get          = 21.4[dC]
X_90_Width        = 8.17[us]
X_Acq_Time        = 0.83361792[s]
X_Angle           = 30[deg]
X_Atn             = 6.5[dB]
X_Pulse           = 2.72333333[us]
Irr_Atn_Dec       = 22.66[dB]
Irr_Atn_Noe       = 22.66[dB]
Irr_Noise         = WALTZ
Irr_Pwidth        = 92[us]
Decoupling        = TRUE
```

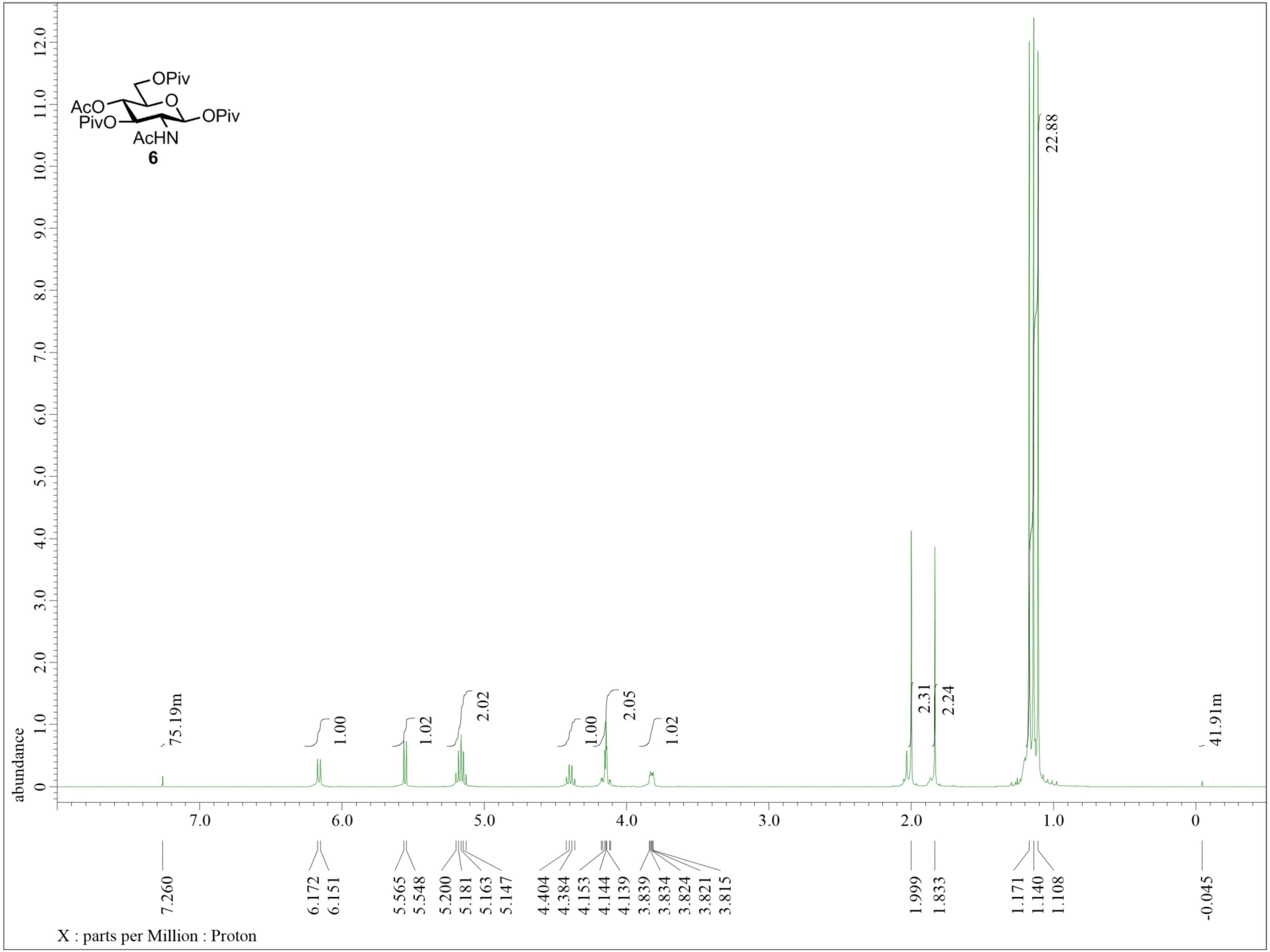

OPiv
AcO
PivO
O
OPiv
AcHN
6
abundance
12.0
11.0
10.0
9.0
8.0
7.0
6.0
5.0
4.0
3.0
2.0
1.0
0
75.19m
1.00
1.02
2.02
1.00
2.05
1.02
2.31
2.24
22.88
41.91m
7.0
6.0
5.0
4.0
3.0
2.0
1.0
0
7.260
6.172
6.151
5.565
5.548
5.200
5.181
5.163
5.147
4.404
4.384
4.153
4.144
4.139
3.839
3.834
3.824
3.821
3.815
1.999
1.833
1.171
1.140
1.108
-0.045
X : parts per Million : Proton

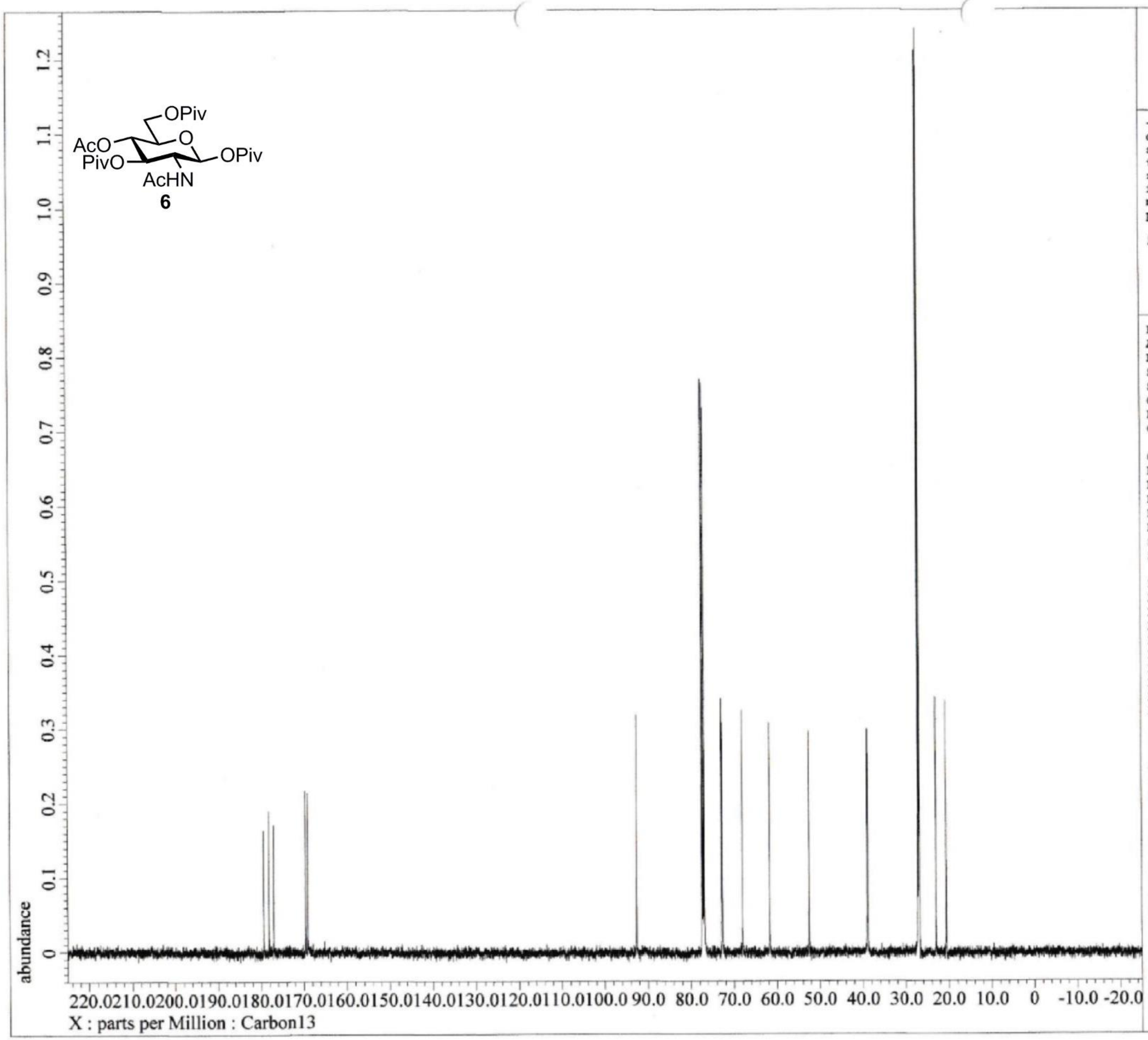

JEOL RESONANCE

---- PROCESSING PARAMETERS ----
dc_balance(0, FALSE)
sexp(2.0[Hz], 0.0[s])
trapezoid(0[%], 0[%], 80[%], 100[%])
zerofill(1)
fft(1, TRUE, TRUE)
machinephase
ppm

以下に由来:: NAc_4Ac_carbon-1-1.jdf

Filename	= NAc_4Ac_carbon-1-2.jdf
Author	= console
Experiment	= carbon.jxp
Sample_Id	= NAc_4Ac
Solvent	= CHLOROFORM-D
Creation_Time	= 17-JUL-2015 15:26:10
Revision_Time	= 17-JUL-2015 15:37:46
Current_Time	= 17-JUL-2015 15:40:02
Comment	= single pulse decoupled gat
Data_Format	= 1D COMPLEX
Dim_Size	= 26214
Dim_Title	= Carbon13
Dim_Units	= [ppm]
Dimensions	= X
Site	= JMN-ECA500
Spectrometer	= DELTA2_NMR
Field_Strength	= 11.7473579[T] (500[MHz])
X_Acq_Duration	= 0.83361792[s]
X_Domain	= 13C
X_Freq	= 125.76529768[MHz]
X_Offset	= 100[ppm]
X_Points	= 32768
X_Prescans	= 4
X_Resolution	= 1.19959034[Hz]
X_Sweep	= 39.3081761[kHz]
X_Sweep_Clipped	= 31.44654088[kHz]
Irr_Domain	= Proton
Irr_Freq	= 500.15991521[MHz]
Irr_Offset	= 5.0[ppm]
Clipped	= FALSE
Scans	= 200
Total_Scans	= 200
Relaxation_Delay	= 2[s]
Recvr_Gain	= 56
Temp_Get	= 22.3[dC]
X_90_Width	= 8.17[us]
X_Acq_Time	= 0.83361792[s]
X_Angle	= 30[deg]
X_Atn	= 6.5[dB]
X_Pulse	= 2.72333333[us]
Irr_Atn_Dec	= 22.66[dB]
Irr_Atn_Noe	= 22.66[dB]
Irr_Noise	= WALTZ
Irr_Pwidth	= 92[us]
Decoupling	= TRUE

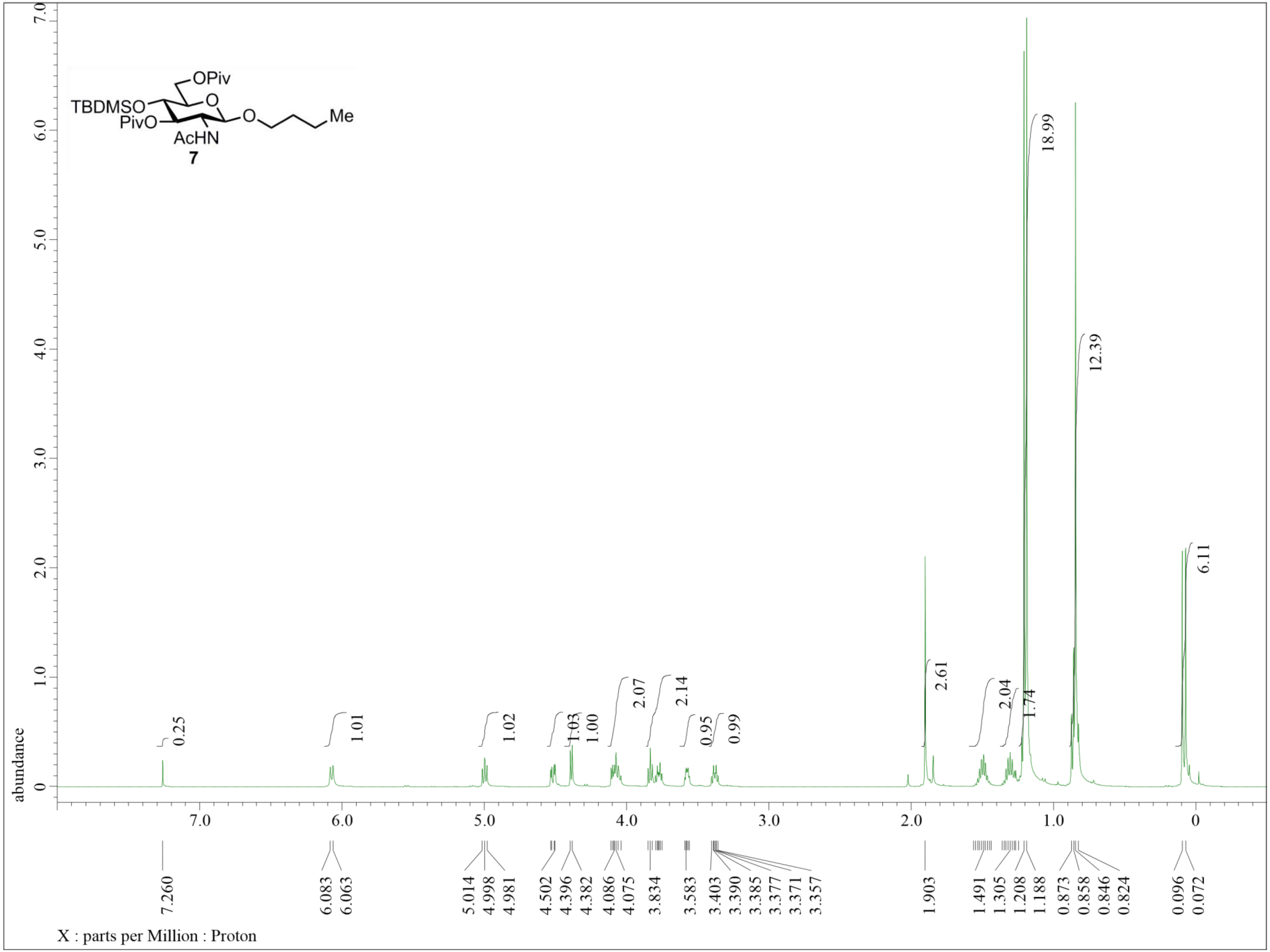

OPiv
TBDMSO
PivO
AcHN
Me
7
18.99
12.39
6.11
2.61
2.04
1.74
0.25
1.01
1.02
1.03
1.00
2.07
2.14
0.95
0.99
abundance
7.260
6.083
6.063
5.014
4.998
4.981
4.502
4.396
4.382
4.086
4.075
3.834
3.583
3.403
3.390
3.385
3.377
3.371
3.357
1.903
1.491
1.305
1.208
1.188
0.873
0.858
0.846
0.824
0.096
0.072
X : parts per Million : Proton

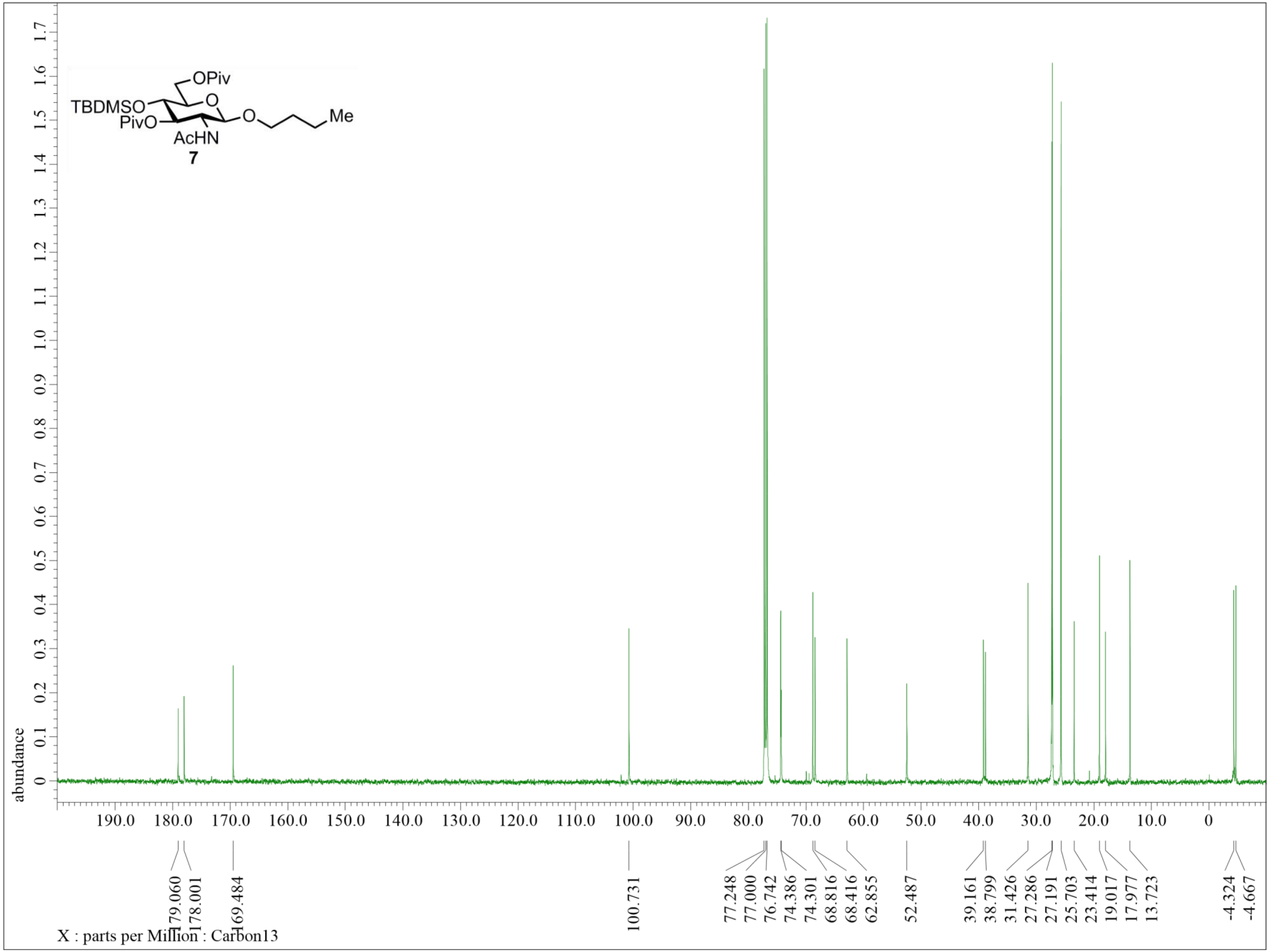

OPiv
TBDMSO
O
PivO
O
Me
AcHN
7
abundance
0
0.1
0.2
0.3
0.4
0.5
0.6
0.7
0.8
0.9
1.0
1.1
1.2
1.3
1.4
1.5
1.6
1.7
190.0
180.0
170.0
160.0
150.0
140.0
130.0
120.0
110.0
100.0
90.0
80.0
70.0
60.0
50.0
40.0
30.0
20.0
10.0
0
179.060
178.001
169.484
100.731
77.248
77.000
76.742
74.386
74.301
68.816
68.416
62.855
52.487
39.161
38.799
31.426
27.286
27.191
25.703
23.414
19.017
17.977
13.723
-4.324
-4.667
X : parts per Million : Carbon13

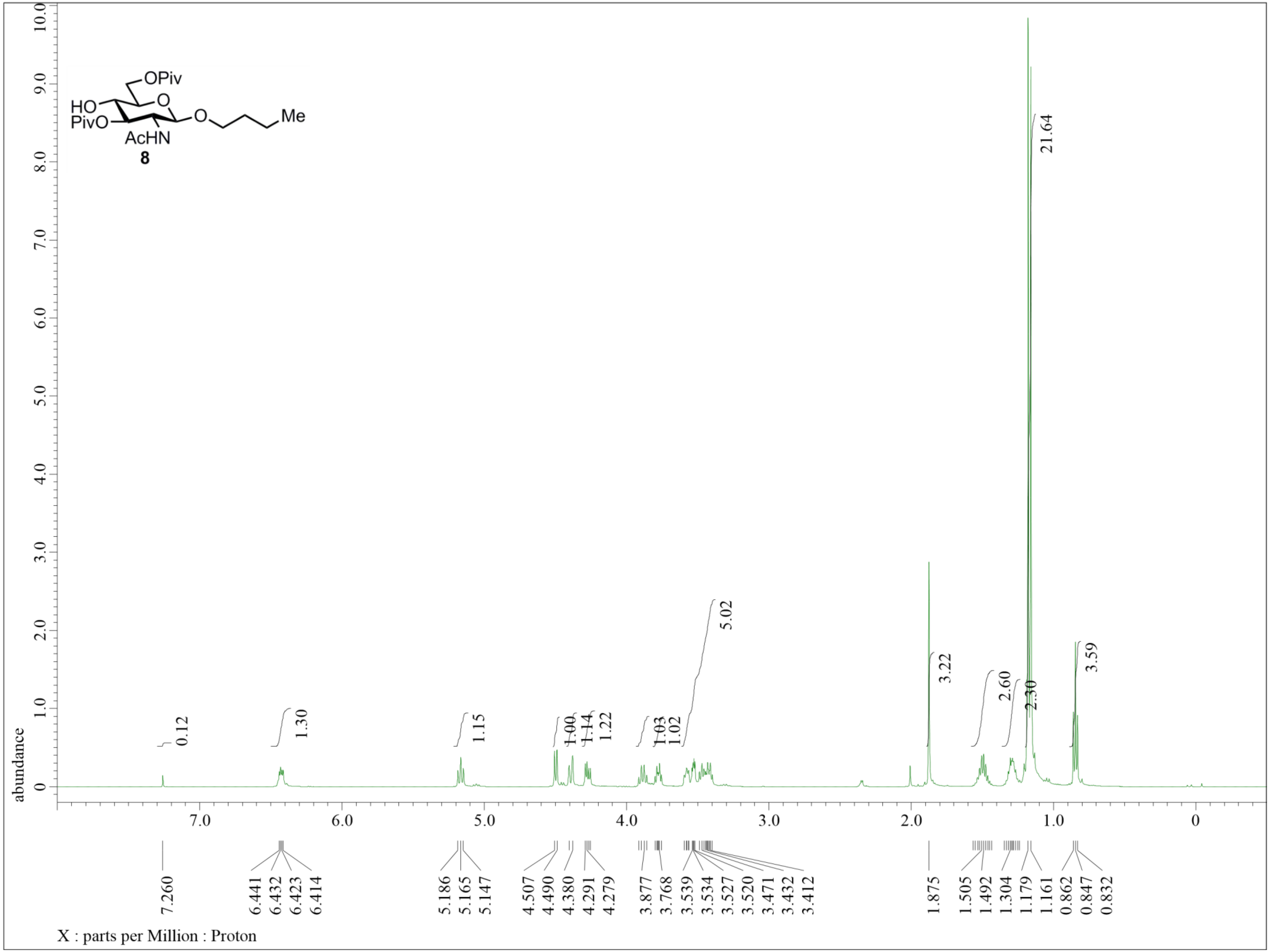
OPiv
HO
PivO
O
O
Me
AcHN
8
abundance
10.0
9.0
8.0
7.0
6.0
5.0
4.0
3.0
2.0
1.0
0
0.12
1.30
1.15
1.00
1.14
1.22
1.03
1.02
5.02
3.22
2.60
2.30
21.64
3.59
7.0
6.0
5.0
4.0
3.0
2.0
1.0
0
7.260
6.441
6.432
6.423
6.414
5.186
5.165
5.147
4.507
4.490
4.380
4.291
4.279
3.877
3.768
3.539
3.534
3.527
3.520
3.471
3.432
3.412
1.875
1.505
1.492
1.304
1.179
1.161
0.862
0.847
0.832
X : parts per Million : Proton

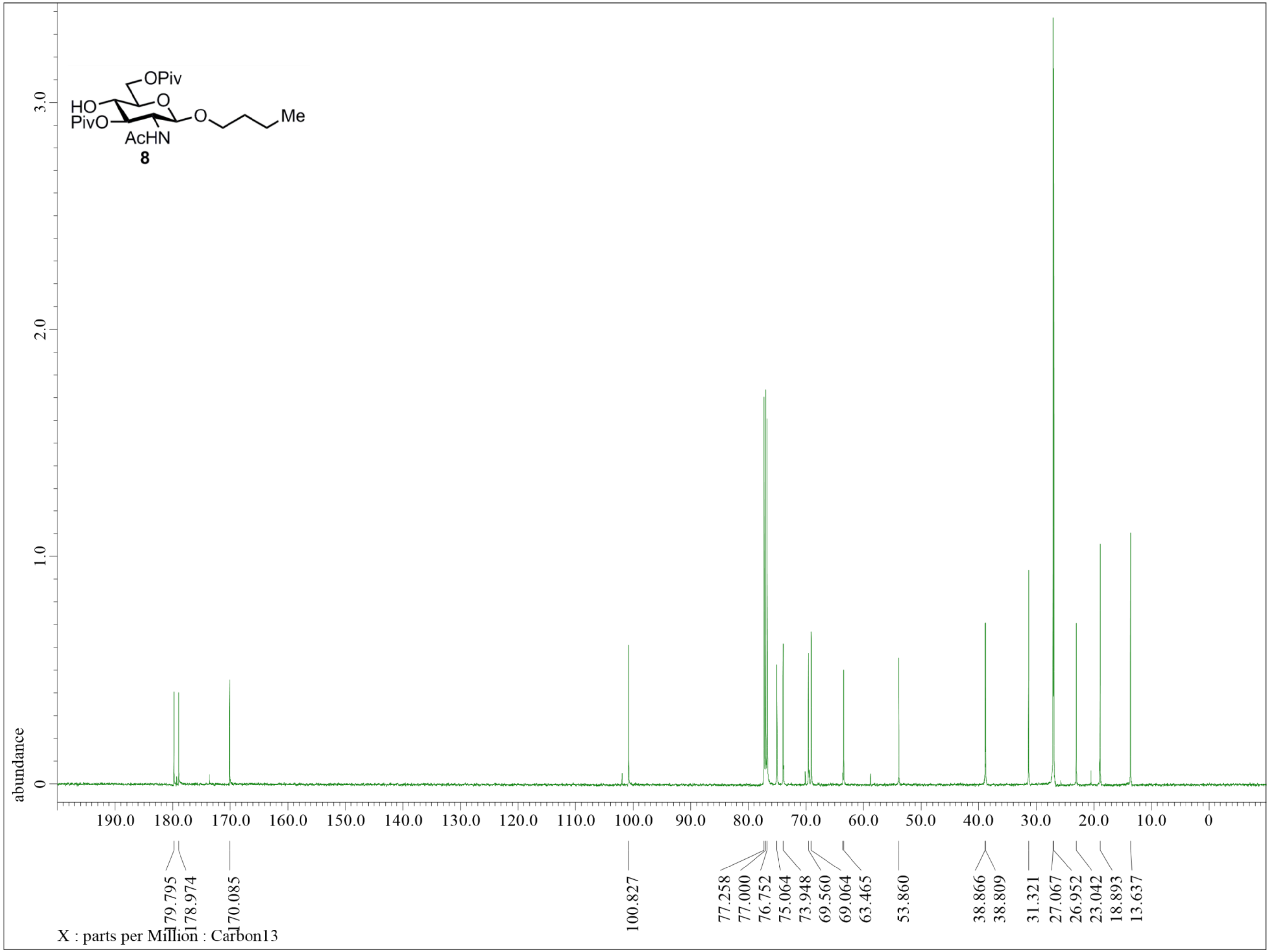

OPiv
HO
O
PivO
O
Me
AcHN
8
3.0
2.0
1.0
0
abundance
190.0
180.0
170.0
160.0
150.0
140.0
130.0
120.0
110.0
100.0
90.0
80.0
70.0
60.0
50.0
40.0
30.0
20.0
10.0
0
179.795
178.974
170.085
100.827
77.258
77.000
76.752
75.064
73.948
69.560
69.064
63.465
53.860
38.866
38.809
31.321
27.067
26.952
23.042
18.893
13.637
X : parts per Million : Carbon13

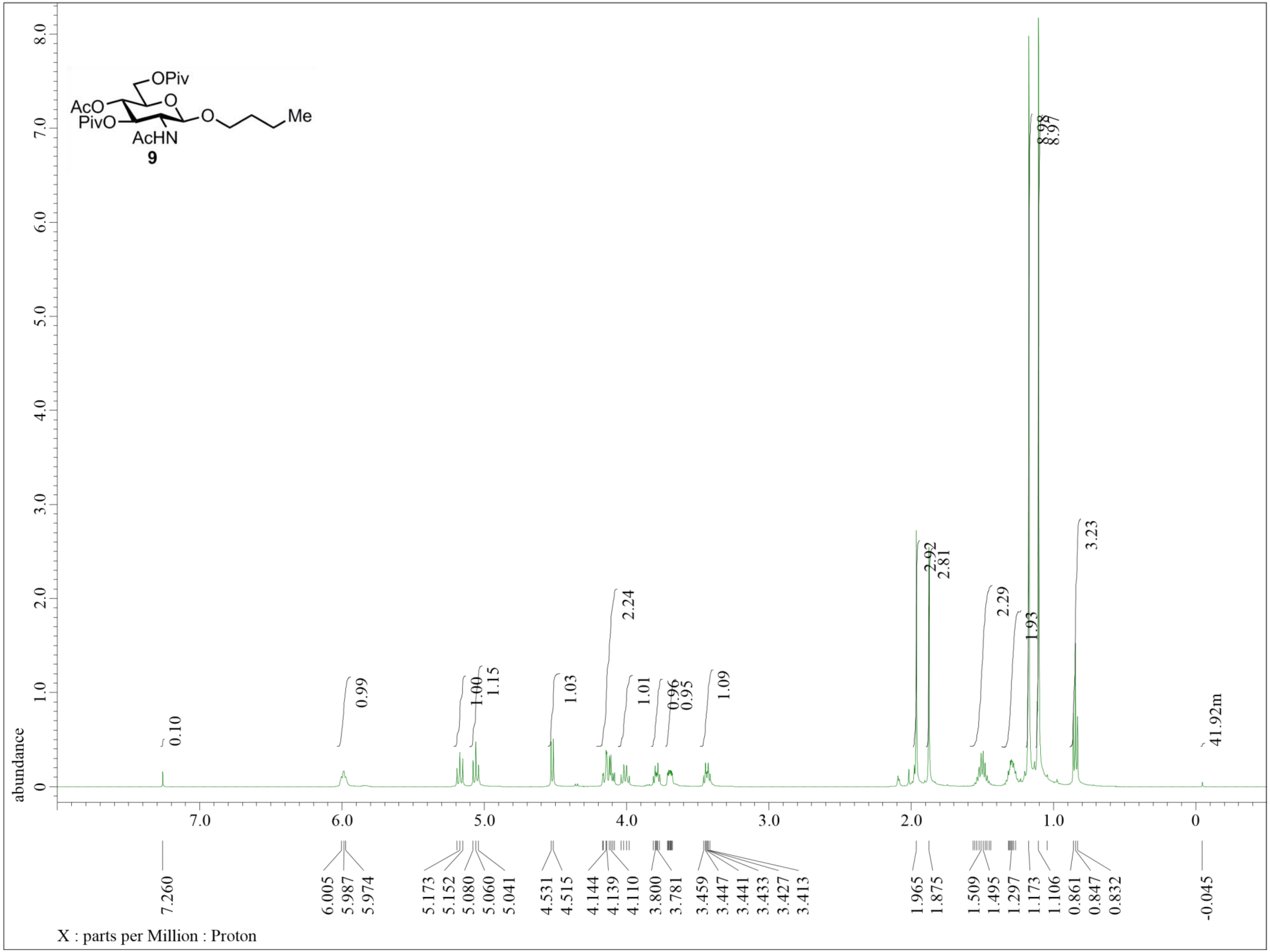
OPiv
AcO
PivO
O
O
Me
AcHN
9
abundance
X : parts per Million : Proton
7.260
6.005
5.987
5.974
5.173
5.152
5.080
5.060
5.041
4.531
4.515
4.144
4.139
4.110
3.800
3.781
3.459
3.447
3.441
3.433
3.427
3.413
1.965
1.875
1.509
1.495
1.297
1.173
1.106
0.861
0.847
0.832
-0.045
0.10
0.99
1.00
1.15
1.03
2.24
1.01
0.96
0.95
1.09
2.92
2.81
2.29
1.93
8.98
8.97
3.23
41.92m

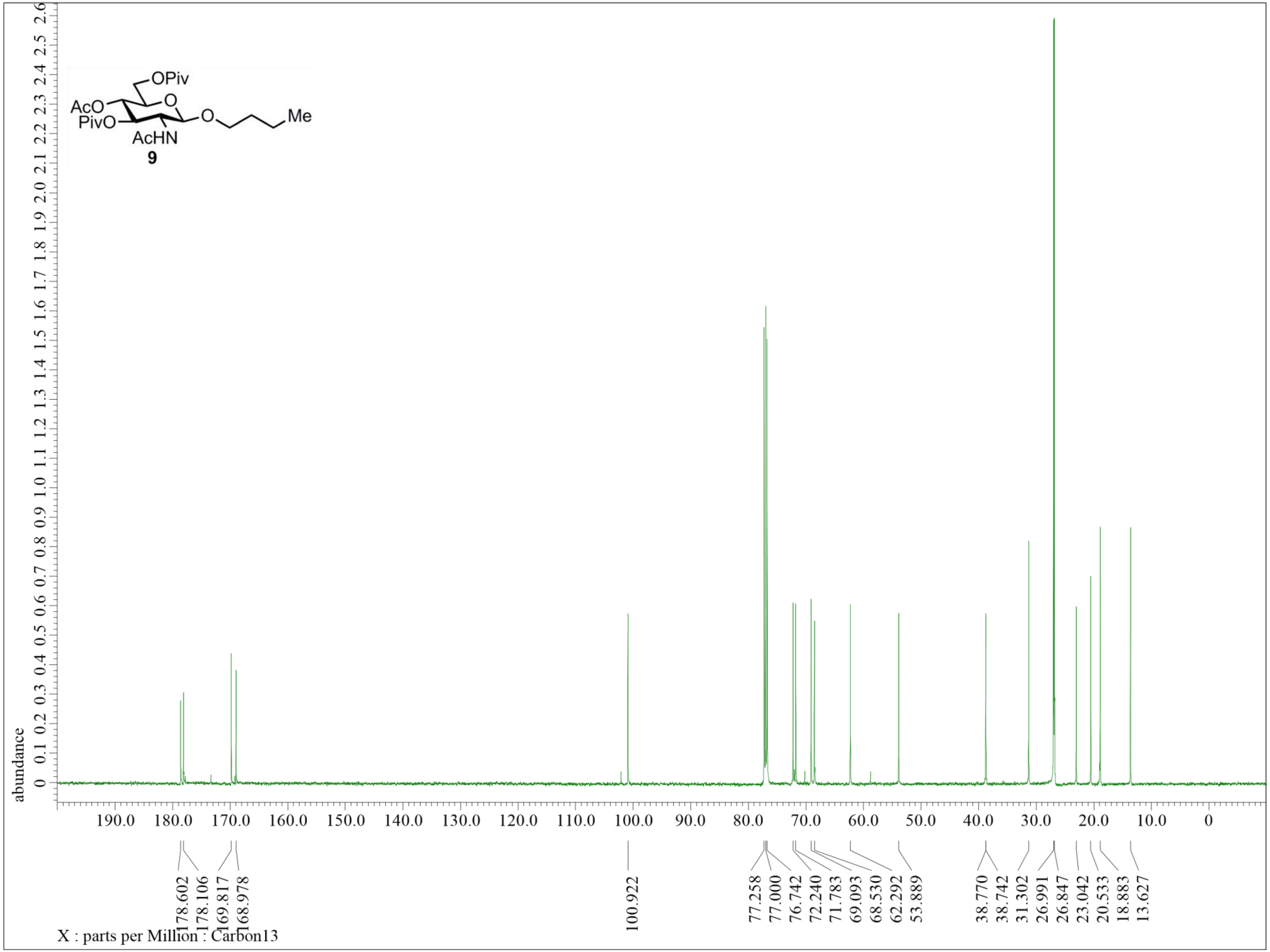
OPiv
AcO
PivO
O
O
Me
AcHN
9
abundance
X : parts per Million : Carbon13
178.602
178.106
169.817
168.978
100.922
77.258
77.000
76.742
72.240
71.783
69.093
68.530
62.292
53.889
38.770
38.742
31.302
26.991
26.847
23.042
20.533
18.883
13.627

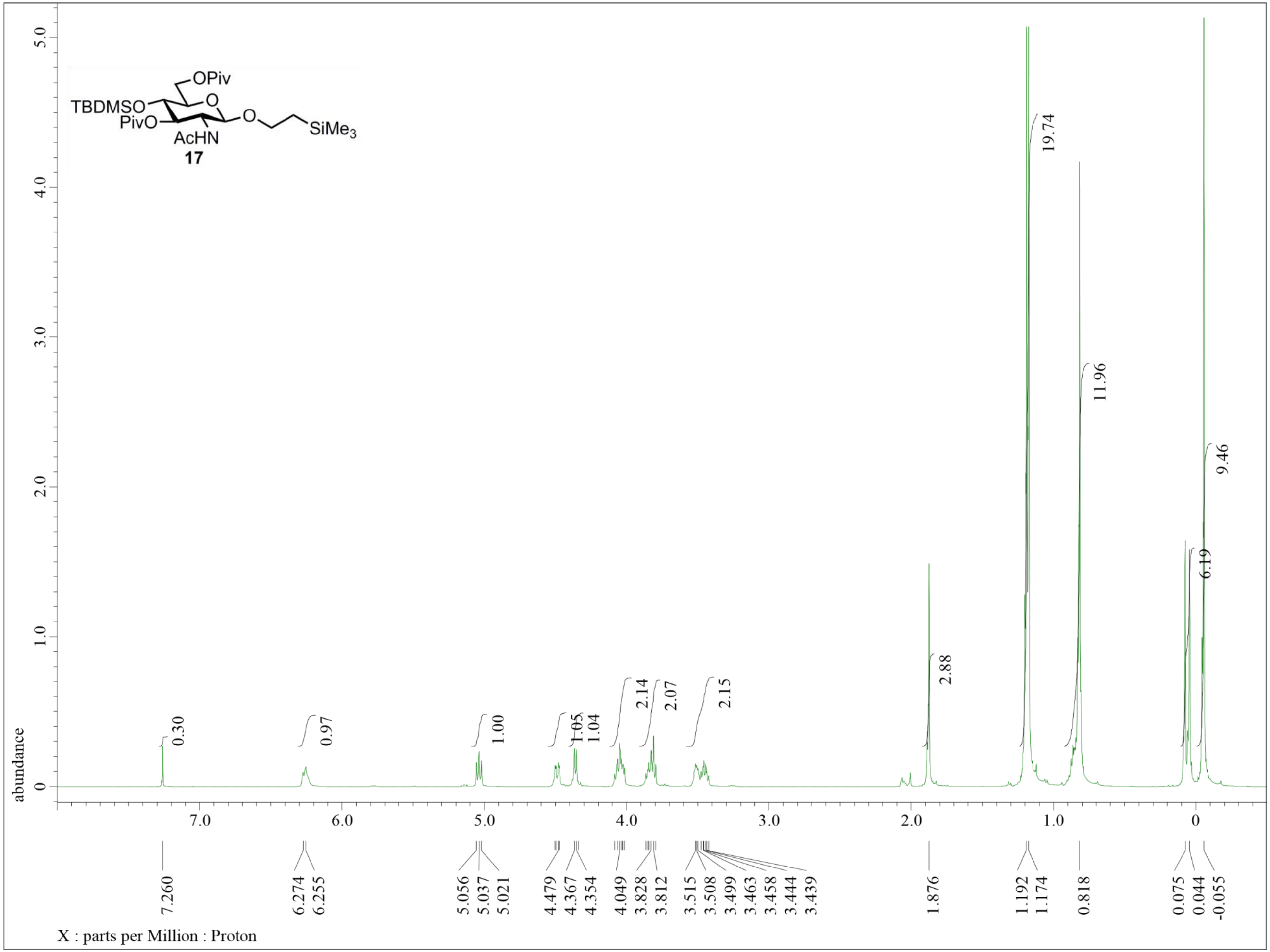

OPiv
TBDMSO
O
PivO
O
AcHN
SiMe3
17
abundance
5.0
4.0
3.0
2.0
1.0
0
0.30
0.97
1.00
1.05
1.04
2.14
2.07
2.15
2.88
19.74
11.96
6.19
9.46
7.0
6.0
5.0
4.0
3.0
2.0
1.0
0
7.260
6.274
6.255
5.056
5.037
5.021
4.479
4.367
4.354
4.049
3.828
3.812
3.515
3.508
3.499
3.463
3.458
3.444
3.439
1.876
1.192
1.174
0.818
0.075
0.044
-0.055
X : parts per Million : Proton

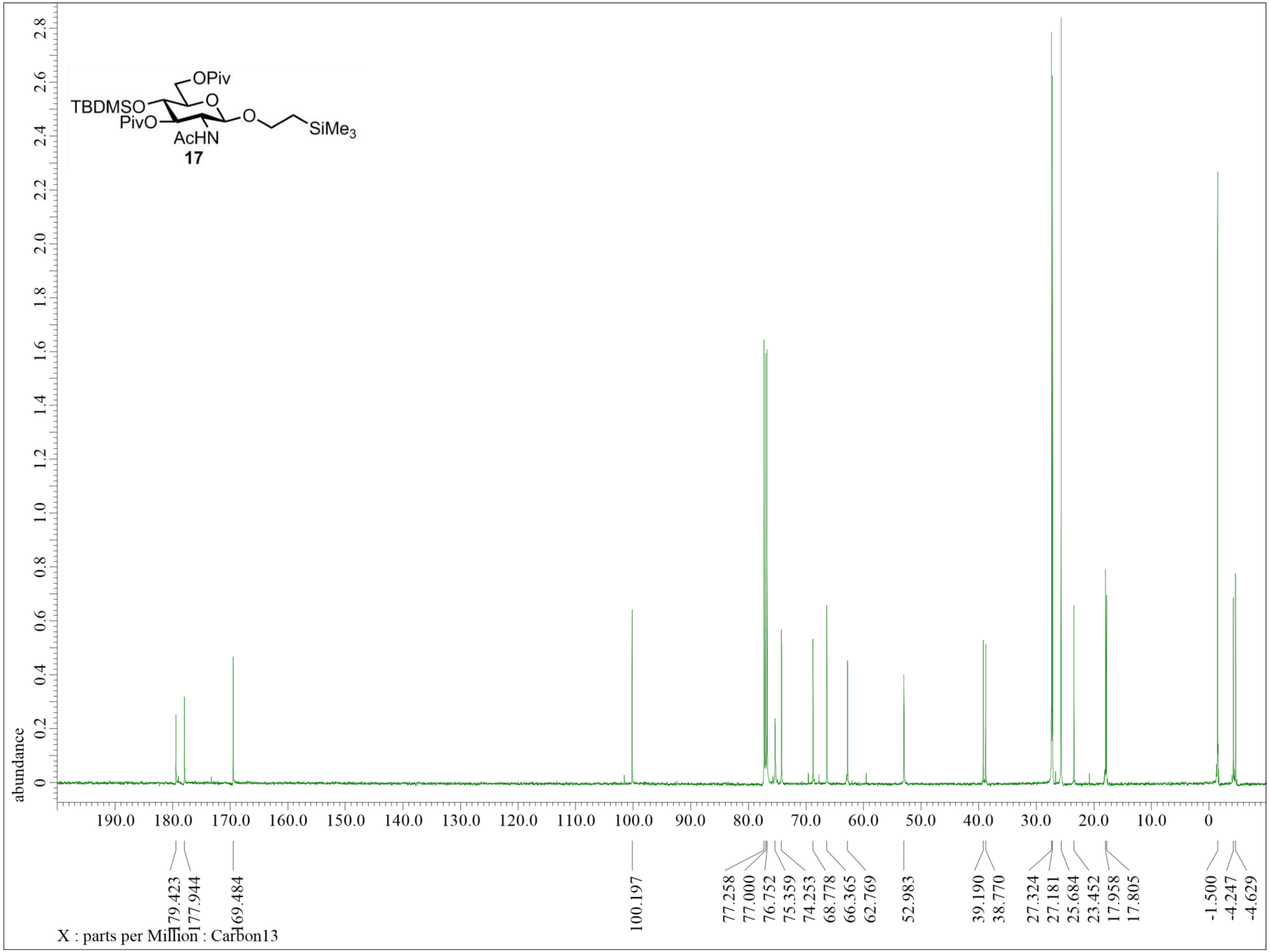

OPiv
TBDMSO
PivO
O
O
SiMe3
AcHN
17
abundance
0 0.2 0.4 0.6 0.8 1.0 1.2 1.4 1.6 1.8 2.0 2.2 2.4 2.6 2.8
190.0 180.0 170.0 160.0 150.0 140.0 130.0 120.0 110.0 100.0 90.0 80.0 70.0 60.0 50.0 40.0 30.0 20.0 10.0 0
179.423
177.944
169.484
100.197
77.258
77.000
76.752
75.359
74.253
68.778
66.365
62.769
52.983
39.190
38.770
27.324
27.181
25.684
23.452
17.958
17.805
-1.500
-4.247
-4.629
X : parts per Million : Carbon13

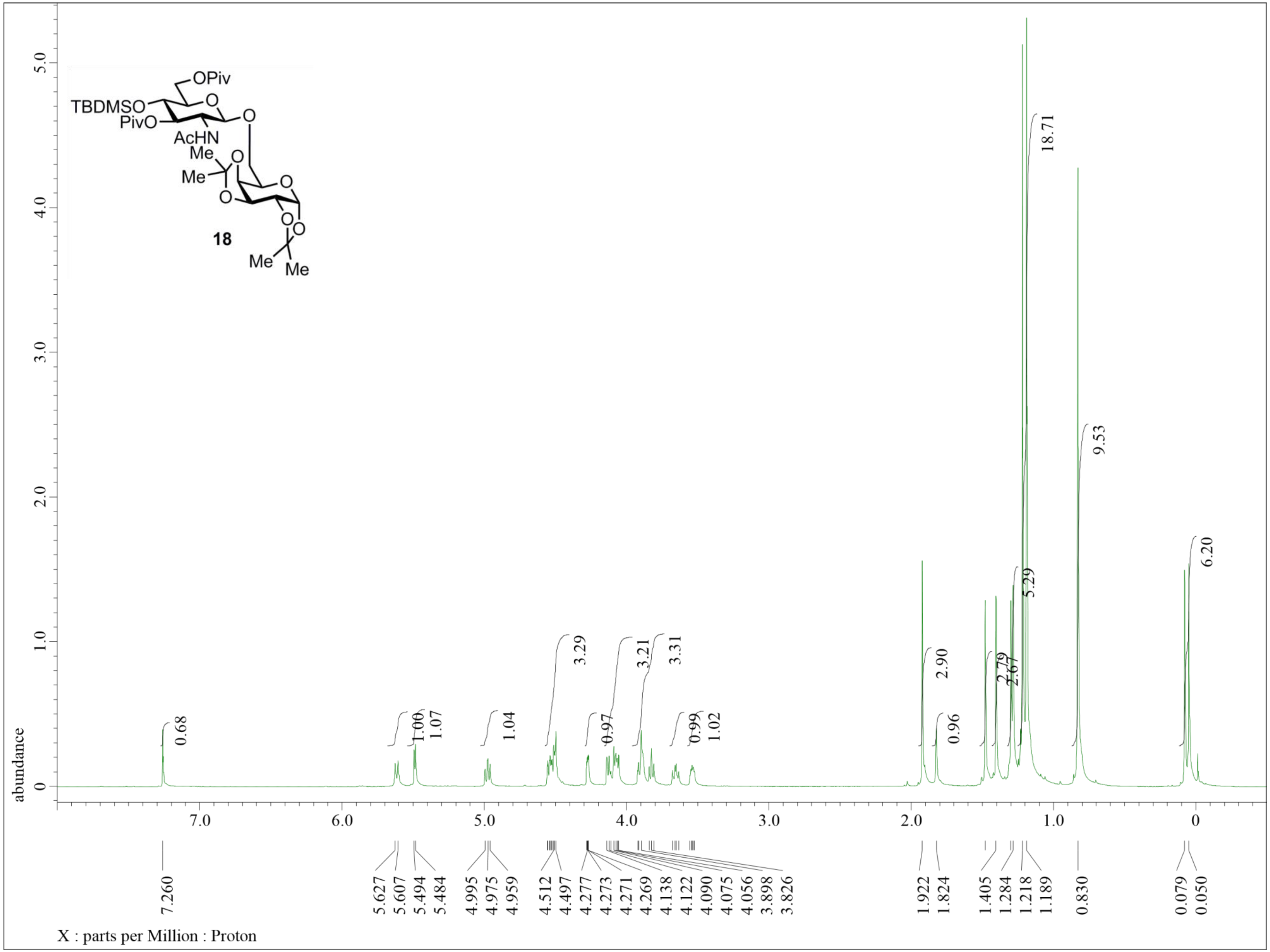
OPiv
TBDMSO
PivO
AcHN
Me
Me
Me
Me
18
abundance
5.0
4.0
3.0
2.0
1.0
0
7.0
6.0
5.0
4.0
3.0
2.0
1.0
0
0.68
1.00
1.07
1.04
3.29
0.97
3.21
3.31
0.99
1.02
2.90
0.96
2.79
2.67
5.29
18.71
9.53
6.20
7.260
5.627
5.607
5.494
5.484
4.995
4.975
4.959
4.512
4.497
4.277
4.273
4.271
4.269
4.138
4.122
4.090
4.075
4.056
3.898
3.826
1.922
1.824
1.405
1.284
1.218
1.189
0.830
0.079
0.050
X : parts per Million : Proton

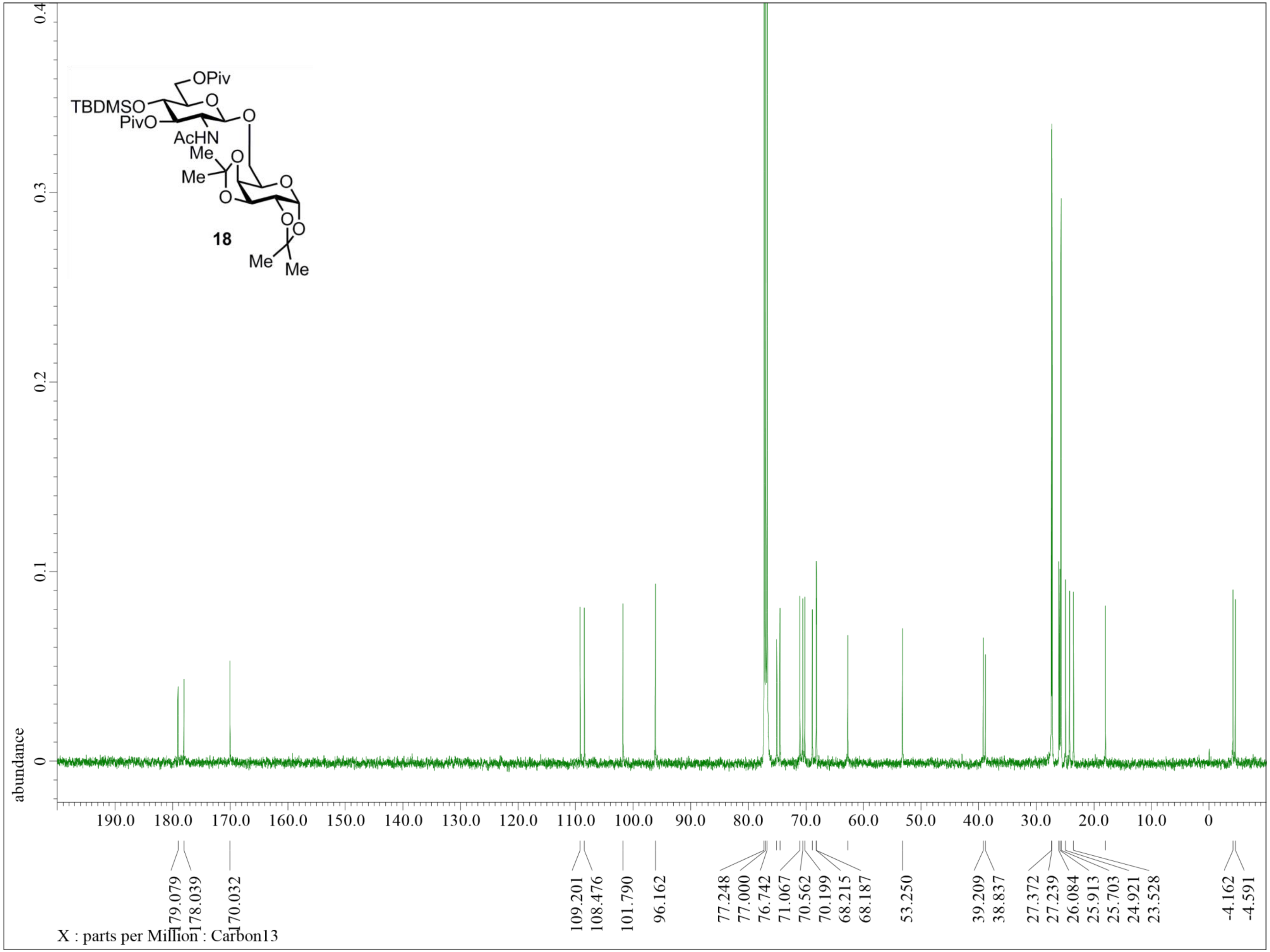

OPiv
TBDMSO
PivO
AcHN
Me
Me
Me
Me
18
abundance
X : parts per Million : Carbon13
179.079
178.039
170.032
109.201
108.476
101.790
96.162
77.248
77.000
76.742
71.067
70.562
70.199
68.215
68.187
53.250
39.209
38.837
27.372
27.239
26.084
25.913
25.703
24.921
23.528
-4.162
-4.591

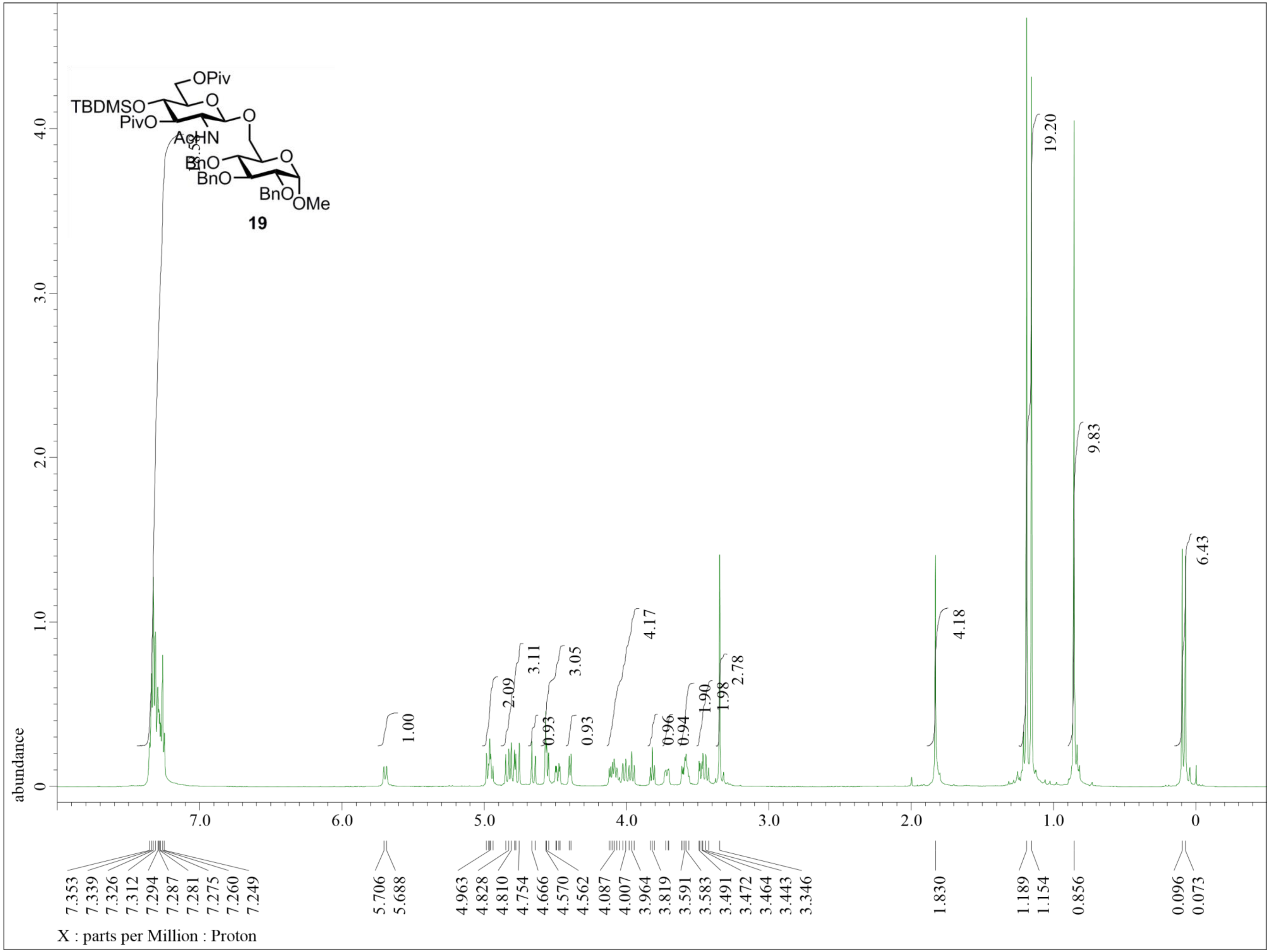
OPiv
TBDMSO
PivO
AcHN
O
BnO
BnO
BnO
OMe
19
abundance
X : parts per Million : Proton
0
1.0
2.0
3.0
4.0
7.0
6.0
5.0
4.0
3.0
2.0
1.0
0
1.00
2.09
3.11
0.93
3.05
0.93
4.17
0.96
0.94
1.90
1.98
2.78
4.18
19.20
9.83
6.43
7.353
7.339
7.326
7.312
7.294
7.287
7.281
7.275
7.260
7.249
5.706
5.688
4.963
4.828
4.810
4.754
4.666
4.570
4.562
4.087
4.007
3.964
3.819
3.591
3.583
3.491
3.472
3.464
3.443
3.346
1.830
1.189
1.154
0.856
0.096
0.073

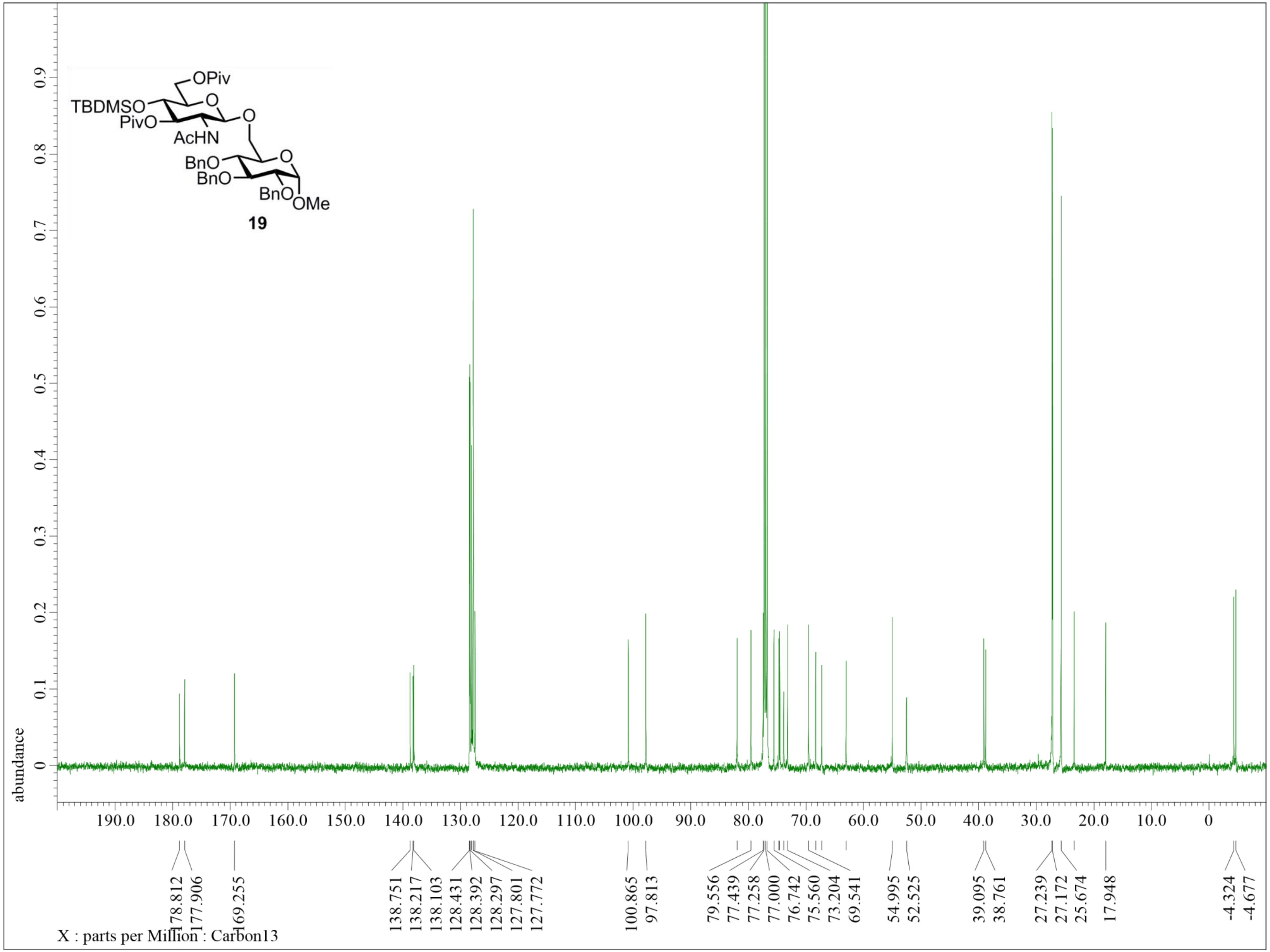
OPiv
TBDMSO
PivO
AcHN
BnO
BnO
BnO
OMe
19
abundance
0.9
0.8
0.7
0.6
0.5
0.4
0.3
0.2
0.1
0
190.0 180.0 170.0 160.0 150.0 140.0 130.0 120.0 110.0 100.0 90.0 80.0 70.0 60.0 50.0 40.0 30.0 20.0 10.0 0
178.812
177.906
169.255
138.751
138.217
138.103
128.431
128.392
128.297
127.801
127.772
100.865
97.813
79.556
77.439
77.258
77.000
76.742
75.560
73.204
69.541
54.995
52.525
39.095
38.761
27.239
27.172
25.674
17.948
-4.324
-4.677
X : parts per Million : Carbon13

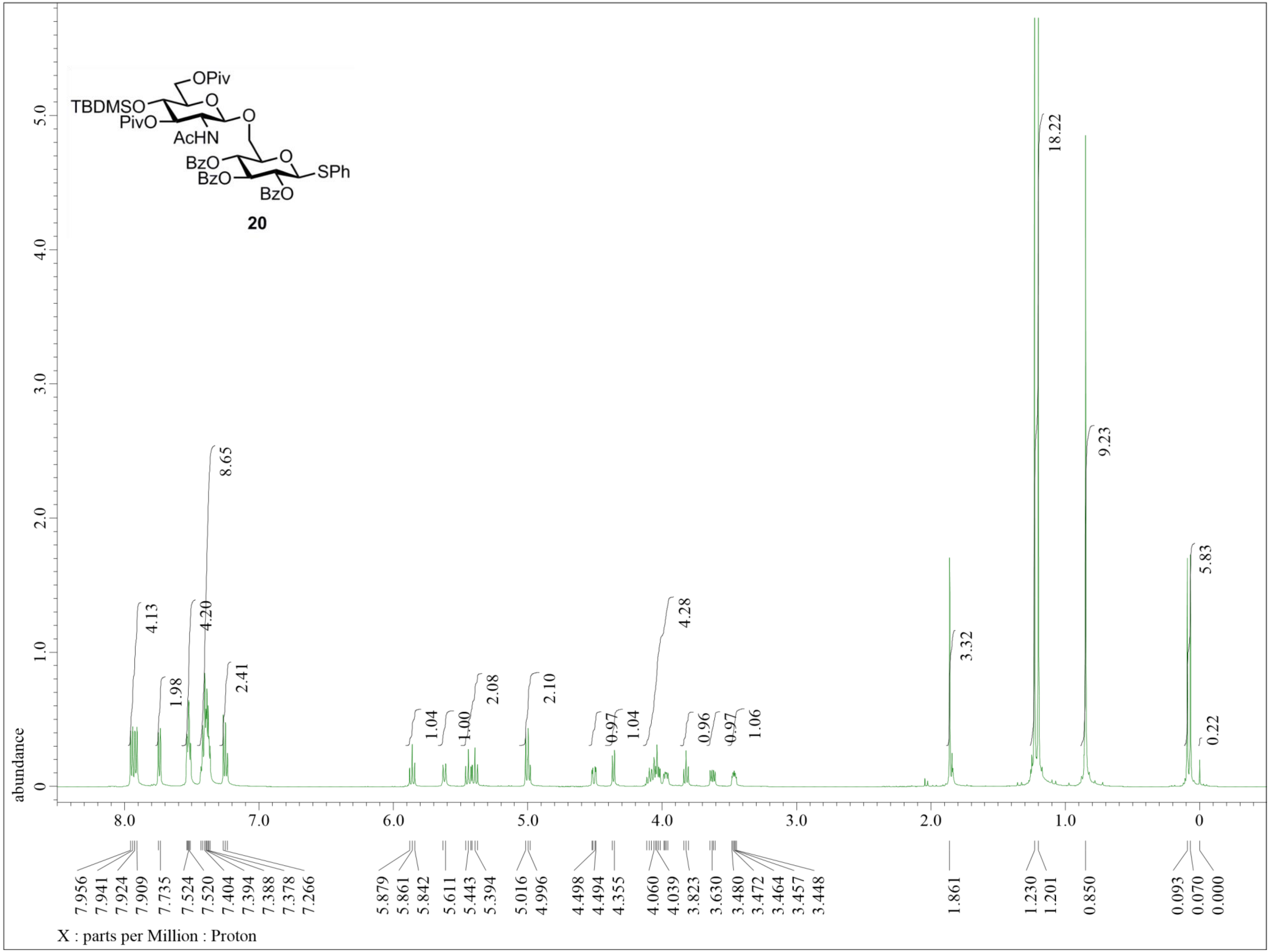
OPiv
TBDMSO
PivO
AcHN
BzO
BzO
BzO
SPh
20
abundance
X : parts per Million : Proton
4.13
1.98
4.20
8.65
2.41
1.04
1.00
2.08
2.10
0.97
1.04
4.28
0.96
0.97
1.06
3.32
18.22
9.23
5.83
0.22
7.956
7.941
7.924
7.909
7.735
7.524
7.520
7.404
7.394
7.388
7.378
7.266
5.879
5.861
5.842
5.611
5.443
5.394
5.016
4.996
4.498
4.494
4.355
4.060
4.039
3.823
3.630
3.480
3.472
3.464
3.457
3.448
1.861
1.230
1.201
0.850
0.093
0.070
0.000

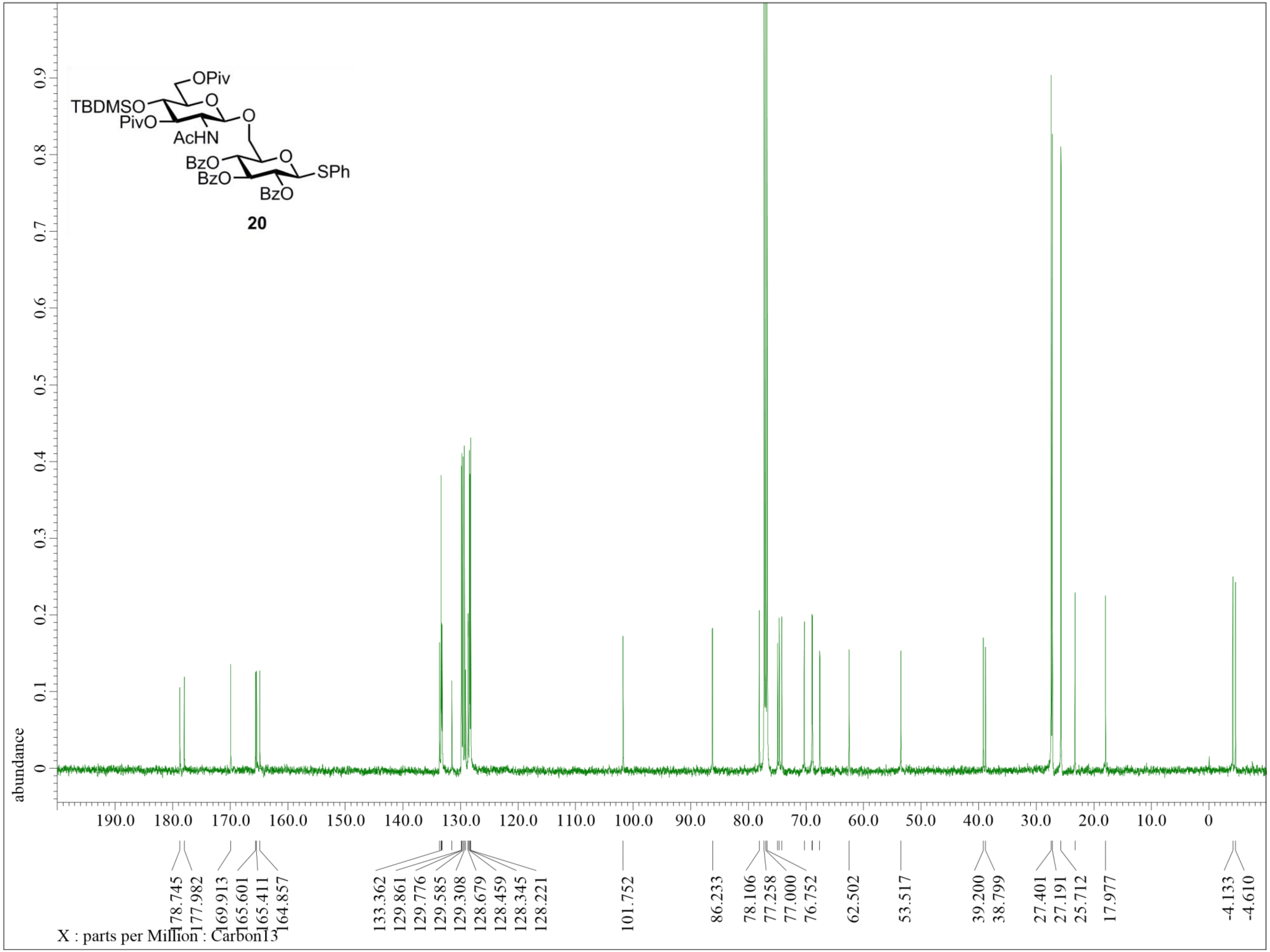

OPiv
TBDMSO
PivO
AcHN
O
BzO
BzO
BzO
SPh
20
abundance
X : parts per Million : Carbon13
178.745
177.982
169.913
165.601
165.411
164.857
133.362
129.861
129.776
129.585
129.308
128.679
128.459
128.345
128.221
101.752
86.233
78.106
77.258
77.000
76.752
62.502
53.517
39.200
38.799
27.401
27.191
25.712
17.977
-4.133
-4.610

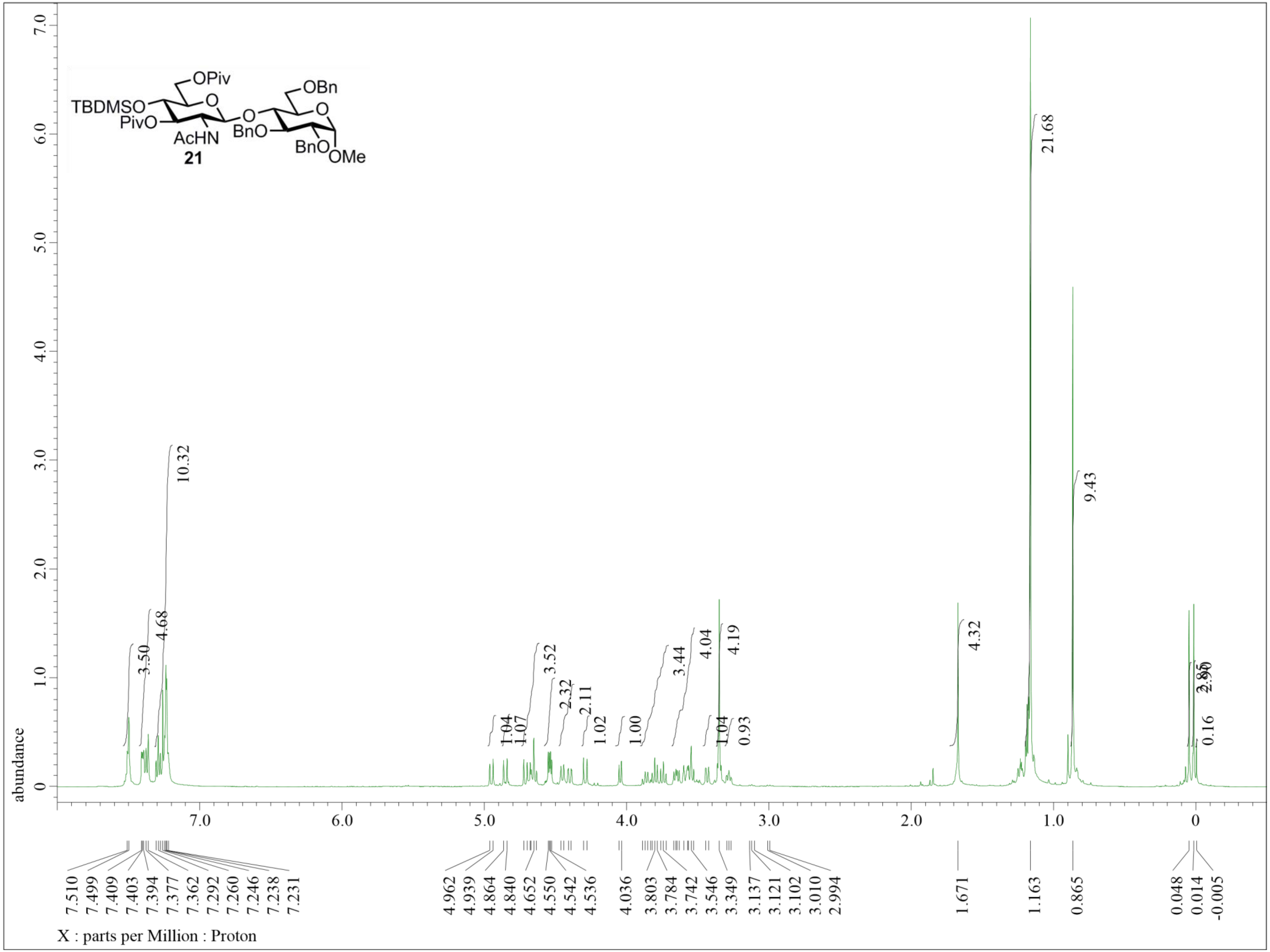

OPiv
TBDMSO
PivO
AcHN
21
O
OBn
BnO
BnO
OMe
abundance
X : parts per Million : Proton
7.0
6.0
5.0
4.0
3.0
2.0
1.0
0
10.32
3.50
4.68
1.04
1.07
3.52
2.32
2.11
1.02
1.00
3.44
4.04
4.19
1.04
0.93
4.32
21.68
9.43
3.85
2.90
0.16
7.510
7.499
7.409
7.403
7.394
7.377
7.362
7.292
7.260
7.246
7.238
7.231
4.962
4.939
4.864
4.840
4.652
4.550
4.542
4.536
4.036
3.803
3.784
3.742
3.546
3.349
3.137
3.121
3.102
3.010
2.994
1.671
1.163
0.865
0.048
0.014
-0.005

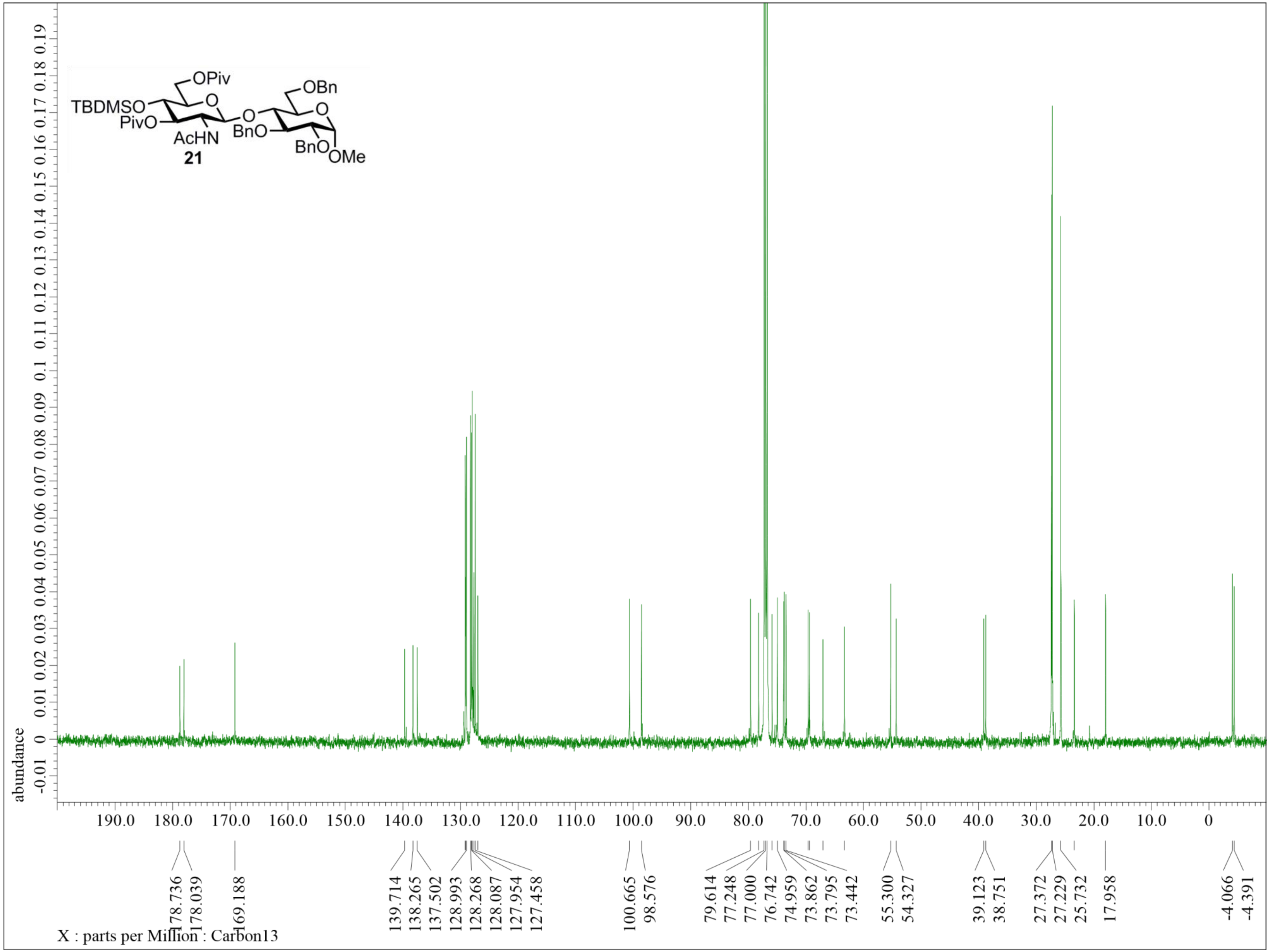

OPiv
TBDMSO
O
PivO
AcHN
21
O
OBn
O
BnO
BnO
OMe
abundance
X : parts per Million : Carbon13
178.736
178.039
169.188
139.714
138.265
137.502
128.993
128.268
128.087
127.954
127.458
100.665
98.576
79.614
77.248
77.000
76.742
74.959
73.862
73.795
73.442
55.300
54.327
39.123
38.751
27.372
27.229
25.732
17.958
-4.066
-4.391

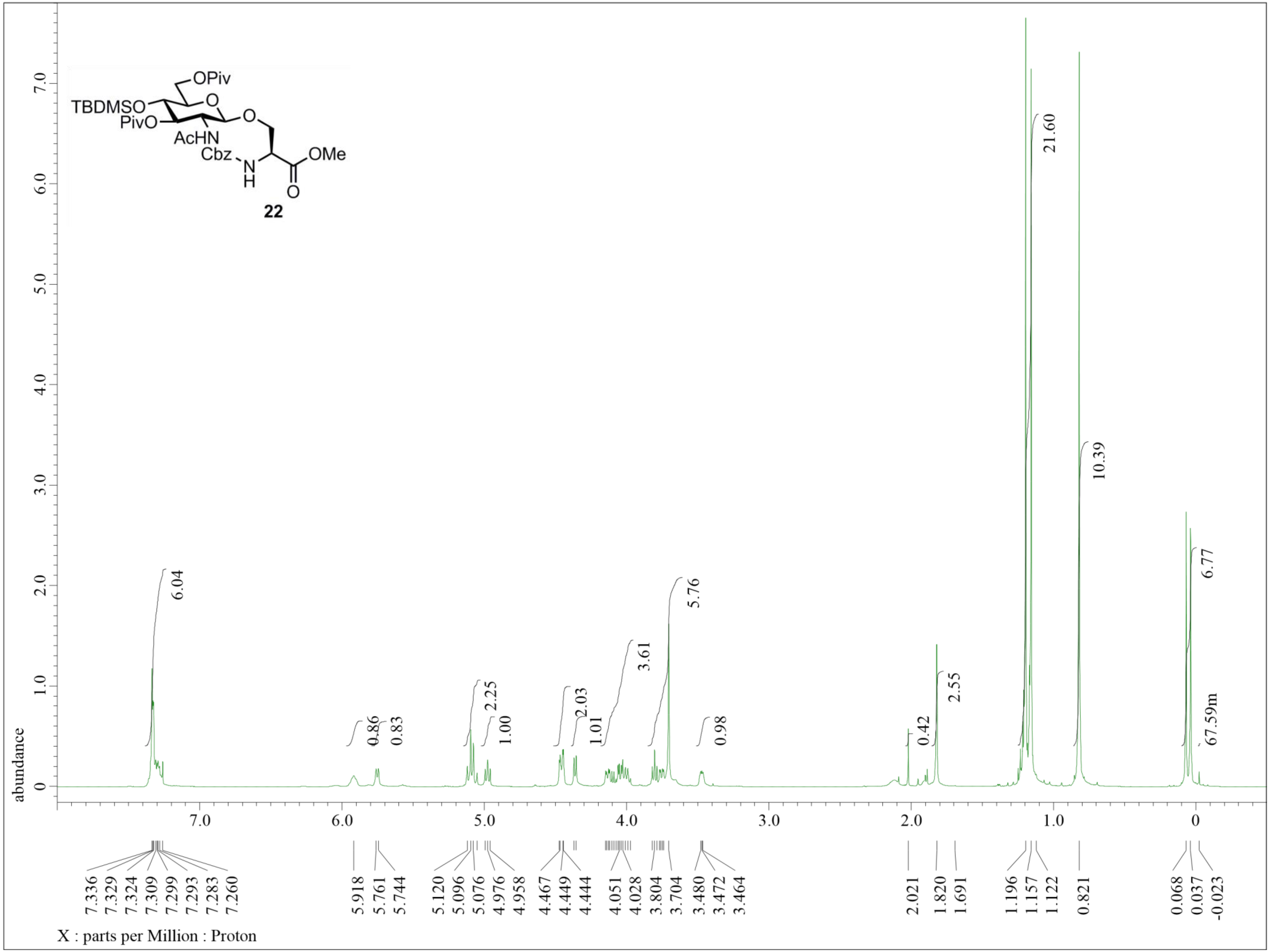

OPiv
TBDMSO
PivO
AcHN
Cbz
N
H
OMe
O
22
abundance
X : parts per Million : Proton
7.0
6.0
5.0
4.0
3.0
2.0
1.0
0
6.04
0.86
0.83
2.25
1.00
2.03
1.01
3.61
5.76
0.98
0.42
2.55
21.60
10.39
6.77
67.59m
7.336
7.329
7.324
7.309
7.299
7.293
7.283
7.260
5.918
5.761
5.744
5.120
5.096
5.076
4.976
4.958
4.467
4.449
4.444
4.051
4.028
3.804
3.704
3.480
3.472
3.464
2.021
1.820
1.691
1.196
1.157
1.122
0.821
0.068
0.037
-0.023

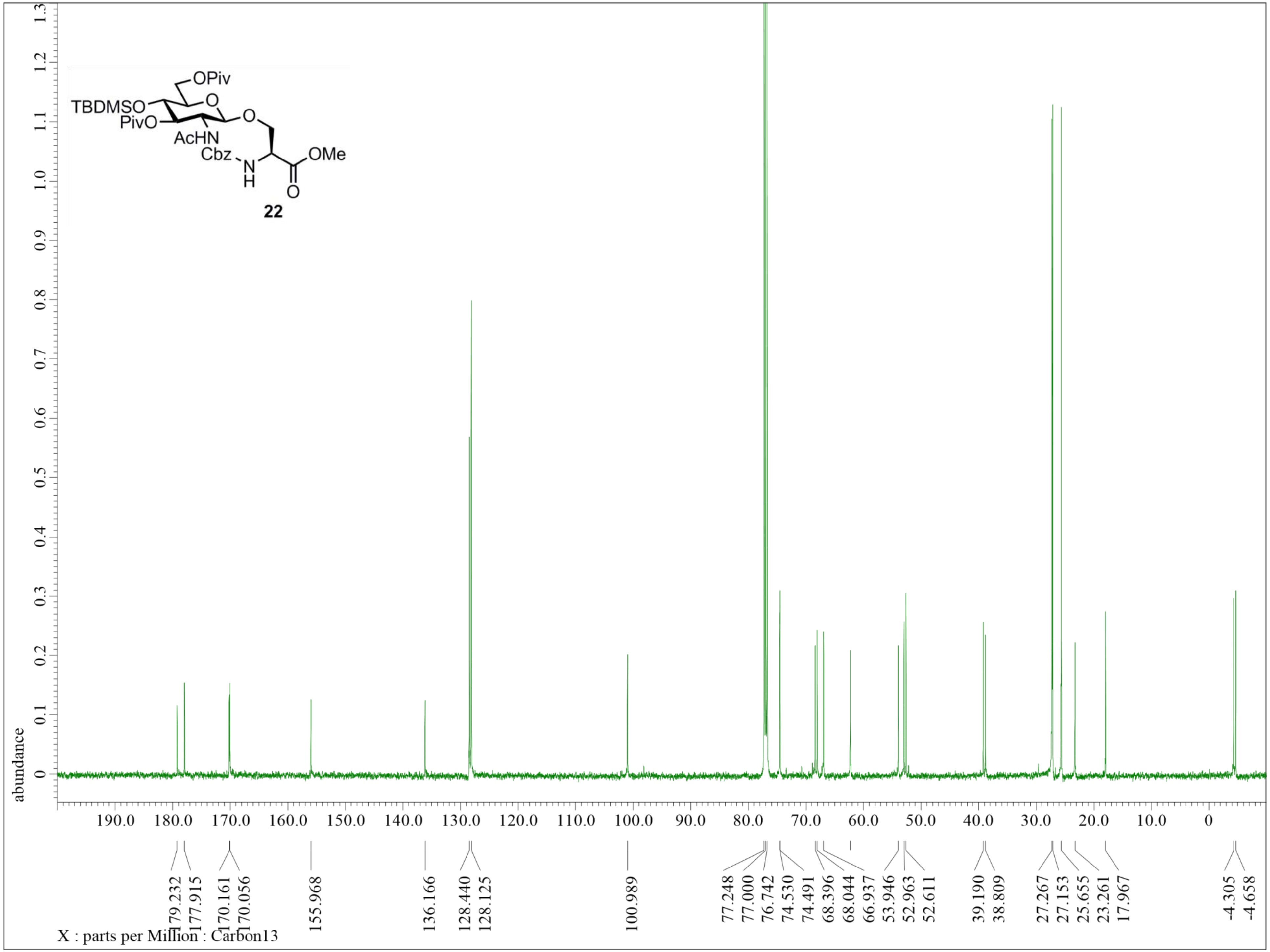
abundance
0 0.1 0.2 0.3 0.4 0.5 0.6 0.7 0.8 0.9 1.0 1.1 1.2 1.3
190.0 180.0 170.0 160.0 150.0 140.0 130.0 120.0 110.0 100.0 90.0 80.0 70.0 60.0 50.0 40.0 30.0 20.0 10.0 0
X : parts per Million : Carbon13
179.232
177.915
170.161
170.056
155.968
136.166
128.440
128.125
100.989
77.248
77.000
76.742
74.530
74.491
68.396
68.044
66.937
53.946
52.963
52.611
39.190
38.809
27.267
27.153
25.655
23.261
17.967
-4.305
-4.658
OPiv
TBDMSO
O
PivO
AcHN
O
Cbz
N
H
OMe
O
22
